# Independent Promoter Recognition by TcpP Precedes Cooperative Promoter Activation by TcpP and ToxR

**DOI:** 10.1128/mBio.02213-21

**Published:** 2021-09-07

**Authors:** A. L. Calkins, L. M. Demey, J. D. Karslake, E. D. Donarski, J. S. Biteen, V. J. DiRita

**Affiliations:** a Department of Chemistry, University of Michigan, Ann Arbor, Michigan, USA; b Department of Microbiology and Molecular Genetics, Michigan State University, East Lansing, Michigan, USA; c Biophysics Program, University of Michigan, Ann Arbor, Michigan, USA; New York University School of Medicine

**Keywords:** gene expression, membrane proteins, single molecule, superresolution

## Abstract

Cholera is a diarrheal disease caused by the Gram-negative bacterium Vibrio cholerae. To reach the surface of intestinal epithelial cells, proliferate, and cause disease, V. cholerae tightly regulates the production of virulence factors such as cholera toxin (*ctxAB*) and the toxin-coregulated pilus (*tcpA-F*). ToxT is directly responsible for regulating these major virulence factors while TcpP and ToxR indirectly regulate virulence factor production by stimulating *toxT* expression. TcpP and ToxR are membrane-localized transcription activators (MLTAs) required to activate *toxT* expression. To gain a deeper understanding of how MLTAs identify promoter DNA while in the membrane, we tracked the dynamics of single TcpP-PAmCherry molecules in live cells using photoactivated localization microscopy and identified heterogeneous diffusion patterns. Our results provide evidence that (i) TcpP exists in three biophysical states (fast diffusion, intermediate diffusion, and slow diffusion), (ii) TcpP transitions between these different diffusion states, (iii) TcpP molecules in the slow diffusion state are interacting with the *toxT* promoter, and (iv) ToxR is not essential for TcpP to localize the *toxT* promoter. These data refine the current model of cooperativity between TcpP and ToxR in stimulating *toxT* expression and demonstrate that TcpP locates the *toxT* promoter independently of ToxR.

## INTRODUCTION

The Gram-negative bacterium Vibrio cholerae infects millions of people each year, causing the diarrheal disease cholera resulting in ∼100,000 deaths annually (1, 2), despite treatments available to combat infection, including vaccines, antibiotic therapy, and oral rehydration therapy (3–10). With changing climate and growing cases of antibiotic-resistant V. cholerae, the number of annual cholera infections is projected to continue to increase (11). Thus, gaining deeper insight into the pathogenesis of V. cholerae will facilitate development of alternative methods of treatment, thereby reducing the global burden of cholera.

Upon ingestion, typically from contaminated water or food, V. cholerae colonizes the crypts of the villi in the distal portion of the small intestine and stimulates production of virulence factors essential for disease progression, such as the toxin-coregulated pilus and cholera toxin (TCP and CtxAB, respectively) (12–17). Transcription of *tcp* and *ctxAB* is directly activated by ToxT (18–21). Expression of *toxT* is highly regulated and positively stimulated by ToxR and TcpP, two membrane-localized transcription activators (MLTAs), which directly bind to the *toxT* promoter (*toxTpro*), with binding sites at −104 to −68 and −55 to −37, respectively (18, 22–28). TcpP and ToxR are bitopic membrane proteins, each containing a cytoplasmic DNA-binding domain (within the PhoB and OmpR families, respectively), a single transmembrane domain, and a periplasmic domain (29). ToxR appears to have an accessory role in *toxT* regulation. Evidence supporting the model that ToxR assists TcpP in *toxT* expression includes that (i) TcpP binds downstream of ToxR, closer than ToxR to the putative RNA polymerase binding site on *toxTpro*, and (ii) overexpression of TcpP results in ToxR-independent *toxT* transcription activation (18, 24, 25, 28). Furthermore, we have previously measured the single-molecule dynamics of TcpP and noted that deletion of *toxR* decreases but does not eliminate the prevalence of TcpP-DNA binding events (30). However, it remains unclear how TcpP and ToxR identify the *toxTpro* from the cytoplasmic membrane.

Signal transduction pathways in prokaryotes consist of one-component and two-component regulatory systems that manage cellular processes in response to extracellular information such as pH, temperature, chemical gradients, and nutrients (31–33). One-component regulatory systems combine their input and output functions in a single protein. MLTAs are a unique family of one-component regulators as they function from the cytoplasmic membrane, whereas the majority (∼97%) of one-component regulators are localized in the cytoplasm (31). These one-component MLTAs like TcpP and ToxR comprise a sensor domain and an output domain that are separated by a transmembrane domain. MLTAs have been experimentally characterized in other, Gram-positive and Gram-negative, pathogenic bacteria and have been shown to regulate genes important for pathogenesis (such as capsule production, acid tolerance, antibiotic resistance, virulence gene regulation, and natural competence) (34–43). Using the Microbial Signal Transduction Database (MIST), we collected candidate MLTAs from 20 bacterial species and found that the prevalence and diversity of MLTAs are much higher than previously anticipated (see Fig. S1 in the supplemental material). These data indicate that MLTAs are more common among bacteria than previously appreciated. Yet, it remains unclear how MLTAs identify a specific promoter(s) while localized to the cytoplasmic membrane. Some challenges emerge in understanding how MLTAs affect their function of activating transcription in response to external stimuli. For example, diffusion of these regulators is constrained to the cytoplasmic membrane. Additionally, the chromosome structure, which is not static, is known to influence the association of an MLTA with its target sequence (44–53). How MLTAs locate their target sequences while bound to the membrane represents a major gap in our knowledge. Here, we investigated the subcellular single-molecule dynamics of TcpP-PAmCherry to understand how TcpP localizes to the *toxTpro* and to develop a general model for how MLTAs identify their DNA targets.

10.1128/mBio.02213-21.1 FIG S1 Maximum likelihood phylogenetic tree of MLTAs collected from the MIST database; phylogenetic tree generated using the TREND server (1, 2). MLTAs displayed here represent a portion of the total MLTAs identified in our small survey. Genus and species information displayed on each branch followed by locus tag and gene designation, where applicable. Numbers next to branch points indicate the bootstrap value. Bootstrap values were generated from 100 replicates. The corresponding MLTA genes are displayed on the right with their predicted domain(s) (in blue) and transmembrane domain(s) (in gray). Download FIG S1, JPG file, 1.0 MB. Copyright © 2021 Calkins et al. 2021 Calkins et al. https://creativecommons.org/licenses/by/4.0/ This content is distributed under the terms of the Creative Commons Attribution 4.0 International license.

Our approach was to apply superresolution single-molecule tracking (SMT) in living cells. Previous work demonstrated that TcpP molecules exhibit heterogeneous diffusion patterns (30, 54). Here, we expand upon this earlier work to study the effect of specific mutations, which alter TcpP binding to DNA or the potential association of TcpP with ToxR, on TcpP subcellular mobility. By tracking the movement of TcpP-PAmCherry molecules within single living V. cholerae cells, we determined the distributions of the heterogeneous motions of TcpP and detected changes in these diffusion coefficients in response to targeted genetic alterations. From these data, we identify three biophysical states (fast diffusion, intermediate diffusion, and slow diffusion), we propose a biological role corresponding to each state, and we suggest an alternative model of *toxT* activation where TcpP independently identifies the *toxTpro* prior to assistance from ToxR.

## RESULTS

### Single-molecule tracking of TcpP-PAmCherry is useful to study promoter identification but cannot probe regulated-intramembrane proteolysis.

To investigate the dynamics of individual TcpP molecules, we generated a V. cholerae strain in which the wild-type (WT) *tcpP* allele is replaced with one expressing TcpP fused at its C terminus to a photoactivatable fluorescent protein, PAmCherry (*tcpP-PAmCherry*). Levels and activity of TcpP are controlled by a two-step proteolytic process known as regulated intramembrane proteolysis (RIP) (55–57). Under RIP-permissive conditions (defined as LB, pH 8.5, 37°C, shaking at 210 rpm) the C terminus of TcpP becomes sensitive to proteolysis by Tsp, a site-1 protease, and YaeL, a site-2 protease; this sensitivity results in the inability of the cell to activate *toxT* expression. Under RIP-nonpermissive conditions (defined as LB, pH 6.5, 30°C, shaking at 110 rpm), TcpP is protected from RIP by TcpH (55–57).

We investigated whether we could assess RIP dynamics using single-molecule tracking. Like wild-type TcpP, TcpP-PAmCherry was sensitive to RIP in the absence of TcpH, indicated by lower levels of TcpP-PAmCherry in *tcpP-PAmCherry*Δ*tcpH* relative to *tcpP-PAmCherry* (see Fig. S2A in the supplemental material). Second, in both *tcpP-PAmCherry* and *tcpP-PAmCherryΔtcpH* a smaller species of TcpP-PAmCherry was observed, referred to as TcpP-PAm* (Fig. S2A). A similar result has been observed for native TcpP in Δ*yaeL* cells and indicates RIP (56). Complementation of *tcpP-PAmCherryΔtcpH* with a plasmid encoding TcpH resulted in a band with the mass of native TcpP (∼29 kDa) (Fig. S3). These data indicate that TcpP-PAmCherry resists RIP in a TcpH-dependent fashion similar to native TcpP. As expected, native TcpP was not detected in the absence of TcpH. These data indicate that (i) TcpP-PAmCherry is sensitive to RIP, (ii) TcpH can protect TcpP-PAmCherry from RIP, and (iii) addition of PAmCherry to the C terminus of TcpP reduces RIP of TcpP-PAmCherry relative to TcpP. These conclusions are supported by similar levels of TcpA, CtxB, and *toxT* expression in *tcpP-PAmCherry* and *tcpP-PAmCherryΔtcpH* (54) (Fig. S2A and Fig. S4). Notwithstanding the detectable levels of TcpP-PAmCherry on immunoblots of total proteins from *tcpP-PAmCherryΔtcpH*, we observed almost no TcpP-PAmCherry molecules in our single-molecule tracking experiments. As a result, we are unable to collect sufficient data to perform any analysis of *tcpP-PAmCherryΔtcpH* cells. Though we cannot determine how RIP influences TcpP-PAmCherry single-molecule dynamics, fusion of PAmCherry to the C terminus of TcpP does not affect its ability to stimulate *toxT* expression (Fig. S4). In addition, activity of TcpP is influenced by homodimerization, mediated by a periplasmic cysteine residue (C207) (58, 59). We sought to determine if addition of PAmCherry to the C terminus of TcpP promotes its ability to dimerize. To test this, we measured *toxT* expression in both *tcpP-PAmCherry* and *tcpPC207S-PAmCherry* cells (Fig. S5). We found that PAmCherry does not compensate for loss of C207, suggesting that it does not stimulate dimerization of TcpP-PAmCherry. These data indicate that PAmCherry does not simulate dimerization of TcpP-PAmCherry. Lastly, addition of PAmCherry to the C terminus of TcpP does not affect the growth rate of V. cholerae (Fig. S6). Therefore, TcpP-PAmCherry is an effective tool to understand how TcpP locates the *toxTpro* from its position in the membrane.

10.1128/mBio.02213-21.2 FIG S2 (A and B) Western blot assays of cultures grown under virulence-inducing conditions for 6 h; see Materials and Methods for primary antibody dilution. Photoactivatable mCherry (PAmCherry) is fused to the C terminus of TcpP and is under the control of its endogenous promoter on the chromosome. Addition of PAmCherry to TcpP results in two species: TcpP-PAmCherry (∼70 kDa) and TcpP-PAmCherry* (∼36 kDa). Deletion of *tcpH* yields lower levels of TcpP-PAmCherry and TcpP-PAmCherry*, likely due to an increase in regulated intramembrane proteolysis (RIP). Download FIG S2, TIF file, 0.6 MB. Copyright © 2021 Calkins et al. 2021 Calkins et al. https://creativecommons.org/licenses/by/4.0/ This content is distributed under the terms of the Creative Commons Attribution 4.0 International license.

10.1128/mBio.02213-21.3 FIG S3 Western blot assays of cultures grown under virulence-inducing conditions for 6 h with or without arabinose; see Materials and Methods for primary antibody dilution. *tcpP-PAmCherry ΔtcpH* cells harbor an arabinose-inducible vector (pBAD18) carrying *tcpH*. Ectopic expression of *tcpH* complemented deletion of *tcpH*. Complementation of *tpcH* also resulted in an additional TcpP band, ∼29 kDa, that corresponds to native TcpP. Download FIG S3, TIF file, 0.04 MB. Copyright © 2021 Calkins et al. 2021 Calkins et al. https://creativecommons.org/licenses/by/4.0/ This content is distributed under the terms of the Creative Commons Attribution 4.0 International license.

10.1128/mBio.02213-21.4 FIG S4 Average *toxT* fold change, relative to WT, across three biological replicates (determined via the ΔΔ*C_T_* method) (61). mRNA was collected from cells after 2 h under virulence-inducing conditions, and error bars represent standard error of the mean. Download FIG S4, TIF file, 0.08 MB. Copyright © 2021 Calkins et al. 2021 Calkins et al. https://creativecommons.org/licenses/by/4.0/ This content is distributed under the terms of the Creative Commons Attribution 4.0 International license.

10.1128/mBio.02213-21.5 FIG S5 *toxT* expression in V. cholerae cells determined using a plasmid-based *toxT*::*GFP* transcriptional reporter. At each time point, *toxT* expression was determined by measuring green fluorescent protein (GFP) fluorescence (excitation, 488 nm, and emission, 515 nm) and optical density (600 nm). The data here are an average from three biological replicates. Error bars represent the standard error of the mean. Download FIG S5, TIF file, 0.02 MB. Copyright © 2021 Calkins et al. 2021 Calkins et al. https://creativecommons.org/licenses/by/4.0/ This content is distributed under the terms of the Creative Commons Attribution 4.0 International license.

10.1128/mBio.02213-21.6 FIG S6 *In vitro* growth curve under virulence-inducing conditions. Optical density (O.D.) values are the average from three biological replicates, and error bars represent standard error of the mean. Download FIG S6, TIF file, 0.1 MB. Copyright © 2021 Calkins et al. 2021 Calkins et al. https://creativecommons.org/licenses/by/4.0/ This content is distributed under the terms of the Creative Commons Attribution 4.0 International license.

### Baseline dynamics of TcpP-PAmCherry.

Single-Molecule Analysis by Unsupervised Gibbs sampling (SMAUG) characterizes the motion of molecules based on the collection of measured displacements (steps) in their single-molecule trajectories. SMAUG estimates the biophysical descriptors of a system by embedding a Gibbs sampler in a Markov chain Monte Carlo framework. This nonparametric Bayesian analysis approach determines the most likely number of mobility states and the average diffusion coefficient of single molecules in each state, the population of each state, and the probability of transitioning between different mobility states over the course of a single trajectory (54). In our previous study, we determined that TcpP-PAmCherry molecules in V. cholerae cells transition between multiple biophysical states: fast diffusion, intermediate diffusion, and slow diffusion (54).

**FIG 1:**
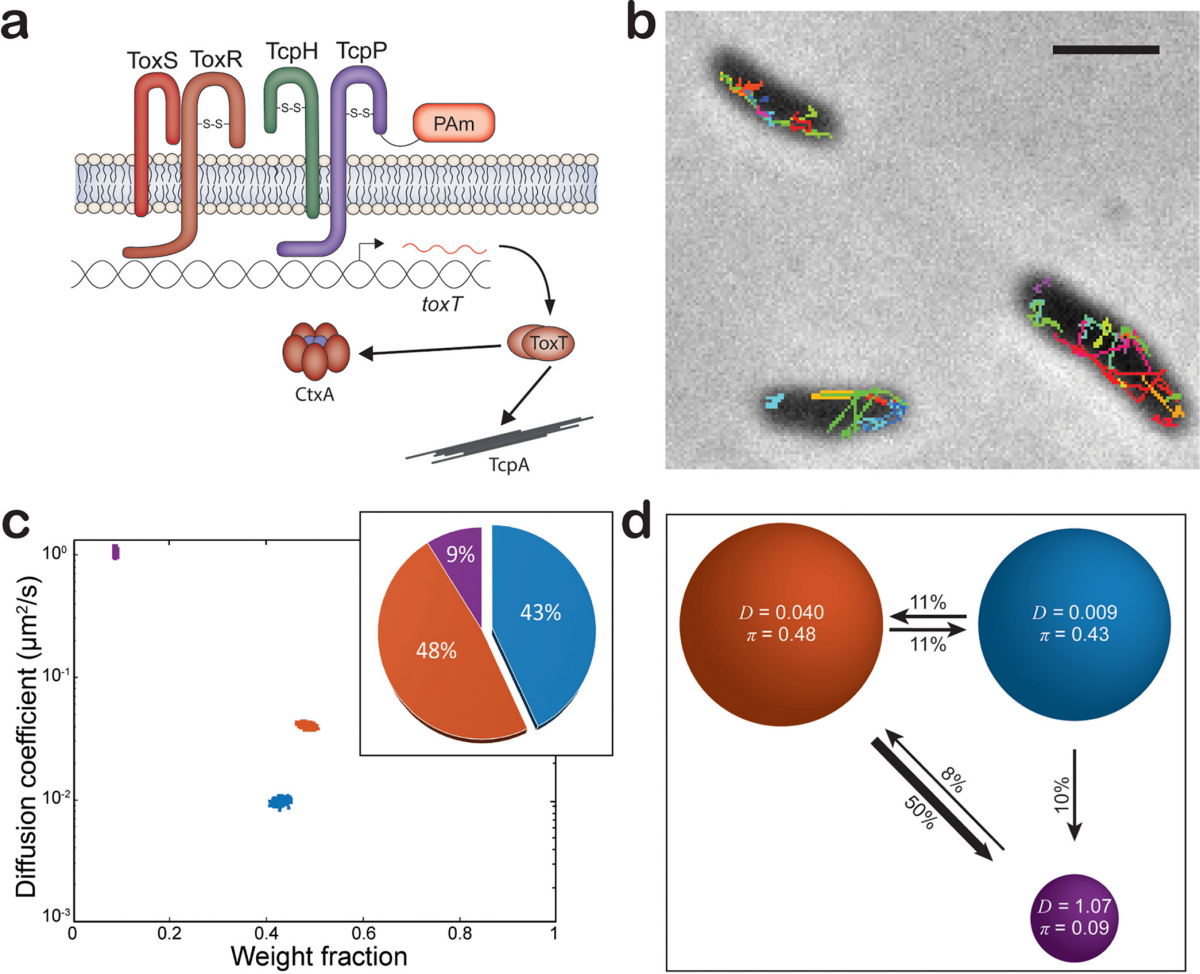
(a) Model of *tcpP-PAmCherry.* (b) Representative single-molecule trajectory maps overlaid on reverse-contrast bright-field image of V. cholerae TcpP-PAmCherry. Only trajectories lasting 0.20 s (5 frames) are shown. Trajectories shown in a variety of colors to show diversity of motion observed. Scale bar, 2 μm. (c) Average single-molecule diffusion coefficients and weight fraction estimates for TcpP-PAmCherry in live V. cholerae cells grown under virulence-inducing conditions. Single-step analysis identifies three distinct diffusion states (fast [purple], intermediate [orange], and slow [blue], respectively). Each point represents the average single-molecule diffusion coefficient versus weight fraction of TcpP-PAmCherry molecules in each distinct mobility state at each saved iteration of the Bayesian algorithm after convergence. The data set contains 54,454 steps from 7,601 trajectories. (Inset) Percentage (weight fraction) of TcpP-PAmCherry in each diffusion state. Colors as in panel. (d) Based on the identification of three distinct diffusion states for TcpP-PAmCherry (three circles with colors as in panel c and with average single-molecule diffusion coefficient, *D*, indicated in μm^2^/s), the average probabilities of transitioning between mobility states at each step are indicated as arrows between those two circles, and the circle areas are proportional to the weight fractions. Low-significance transition probabilities less than 4% are not displayed; for instance, the probability of TcpP-PAmCherry molecules transitioning from the fast diffusion state to the slow diffusion state is 1%. Numbers above the arrows indicate the probability of transition.

Here, we collected a new robust set of TcpP-PAmCherry tracking data in living V. cholerae cells (54,454 steps collected from 7,601 trajectories) to further refine our analysis and to assign biochemical mechanisms to these biophysical observations (a sample of these tracks is shown in Fig. 1b; see also Video S1 at https://doi.org/10.5281/zenodo.5222485). Consistent with our previous results, we ascertained that TcpP-PAmCherry exists in three distinct states (slow diffusion, intermediate diffusion, and fast diffusion; blue, orange, and purple, respectively, in Fig. 1c). Furthermore, we determined that TcpP-PAmCherry molecules do not freely transition between all the diffusion states: we observe that TcpP-PAmCherry molecules can transition between the fast state (purple) and the intermediate state (orange) and between the intermediate state (orange) and the slow state (blue) freely, but there is no significant probability of transitions directly from the fast diffusion state (purple) to the slow diffusion state (blue) on successive steps (Fig. 1d). Thus, the intermediate diffusion state represents a critical biochemical intermediate between the slow and fast diffusion states.

The high transition probability of TcpP-PAmCherry molecules from the intermediate diffusion state to the fast diffusion state (50%) is unexpected, as the fast diffusion state represents the smallest population of TcpP-PAmCherry molecules (9%), with a low probability (8%) of TcpP-PAmCherry molecules transitioning from the fast diffusion state back to the intermediate diffusion state (Fig. 1d). While we cannot directly determine how RIP influences the dynamics of TcpP-PAmCherry, the stark difference in the transition probabilities and the populations of TcpP-PAmCherry in the fast and intermediate diffusion states suggests that fast-diffusing TcpP-PAmCherry molecules are potentially sensitive to some form of degradation.

Given this baseline for the dynamics of TcpP-PAmCherry, we hypothesize that (i) the three diffusion states (slow, intermediate, and fast) are features of TcpP-PAmCherry molecules with three biologically distinct roles; (ii) the slow diffusion state is occupied by TcpP-PAmCherry molecules interacting with DNA, such as *toxTpro*; and (iii) the intermediate diffusion state is influenced by ToxR. We further explore these three hypotheses with V. cholerae mutants below.

### Mutation of the *toxTpro* decreases the slow diffusion state occupancy.

**FIG 2:**
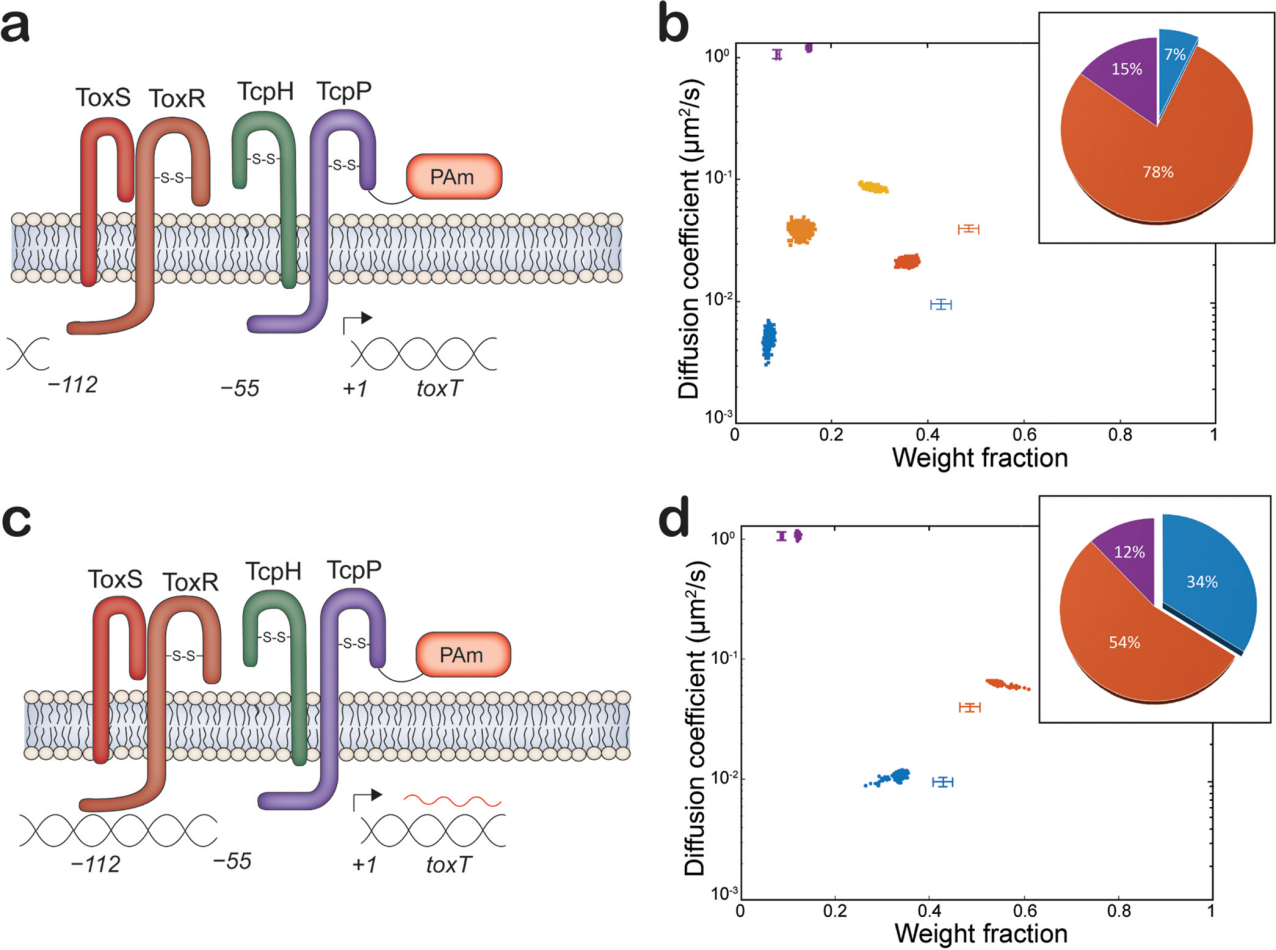
TcpP-PAmCherry diffusion dynamics within live V. cholerae cells containing mutated regions of the *toxT* promoter (*toxTpro*). (a and c) Model of *toxTpro* mutations in *tcpP-PAmCherry toxTpro*Δ(−112–+1), and *tcpP-PAmCherry toxTpro*Δ(−55–+1), respectively. (b and d) Average single-molecule diffusion coefficients and weight fraction estimates for TcpP-PAmCherry in live V. cholerae *tcpP-PAmCherry toxTpro*Δ(−112–+1) (b) and V. cholerae *tcpP-PAmCherry toxTpro*Δ(−55–+1) (d) grown under virulence-inducing conditions. Single-step analysis identifies five and three distinct diffusion states (fast [purple], intermediate [orange, light orange, and yellow], and slow [blue], respectively). Each point represents the average single-molecule diffusion coefficient versus weight fraction of TcpP-PAmCherry molecules in each distinct mobility state at each saved iteration of the Bayesian algorithm after convergence. The data set contains 104,341 steps from 21,274 trajectories for panel b and 75,841 steps from 11,624 trajectories for panel d. The data for TcpP-PAmCherry diffusion in wild-type V. cholerae cells (Fig. 1c) are provided for reference (cross-hairs). (Insets) Percentage (weight fraction) of TcpP-PAmCherry in each diffusion state. Colors as in panel.

We hypothesized that the slow TcpP-PAmCherry diffusion state encompasses molecules specifically interacting with DNA at its binding site in the *toxTpro*. The molecular weight of chromosomal DNA (chromosome 1, 2.96 Mbp) is much higher than that of any protein. Thus, binding of TcpP-PAmCherry to this promoter on the chromosome should result in an extremely low apparent diffusion rate. To test our hypothesis, we removed key binding sites for TcpP (−55 to −37) and both ToxR and TcpP (−112 to +1) in the *toxTpro*, generating *tcpP-PAmCherry toxTpro*Δ(−55–+1) and *tcpP-PAmCherry toxTpro*Δ(−112–+1) (Fig. 2), both of which resulted in a drastic reduction in TcpA production, similar to that of a *ΔtcpP* mutant (Fig. S2A). *toxT* expression was reduced in *tcpP-PAmCherry toxTpro*Δ(−112–+1) but not in *tcpP-PAmCherry toxTpro*Δ(−55–+1) (Fig. S4). It is possible that the *toxTpro*Δ(−55–+1) mutation causes TcpP-PAmCherry and ToxR to stimulate expression of a nonfunctional *toxT* mRNA. Regardless, loss of either region of the *toxTpro* results in loss of production of the TcpA virulence factor.

Relative to the wild type (Fig. 1), deleting both the ToxR and TcpP binding sites [*toxTpro*Δ(−112–+1)] reduces the percentage of slow-diffusing TcpP-PAmCherry to very low levels (7%; Fig. 2b). Thus, TcpP-PAmCherry in the slow diffusion state requires *toxTpro*; therefore, we propose molecules in this state are bound to *toxTpro*. On the other hand, loss of the TcpP binding site alone [*toxTpro*Δ(−55–+1)] reduces the percentage of slow TcpP-PAmCherry molecules only subtly (from 43% to 34%; Fig. 2d). This result is consistent with earlier observations demonstrating that association with ToxR can restore the function of TcpP variants otherwise unable to bind the *toxTpro* (18, 24).

**FIG 3:**
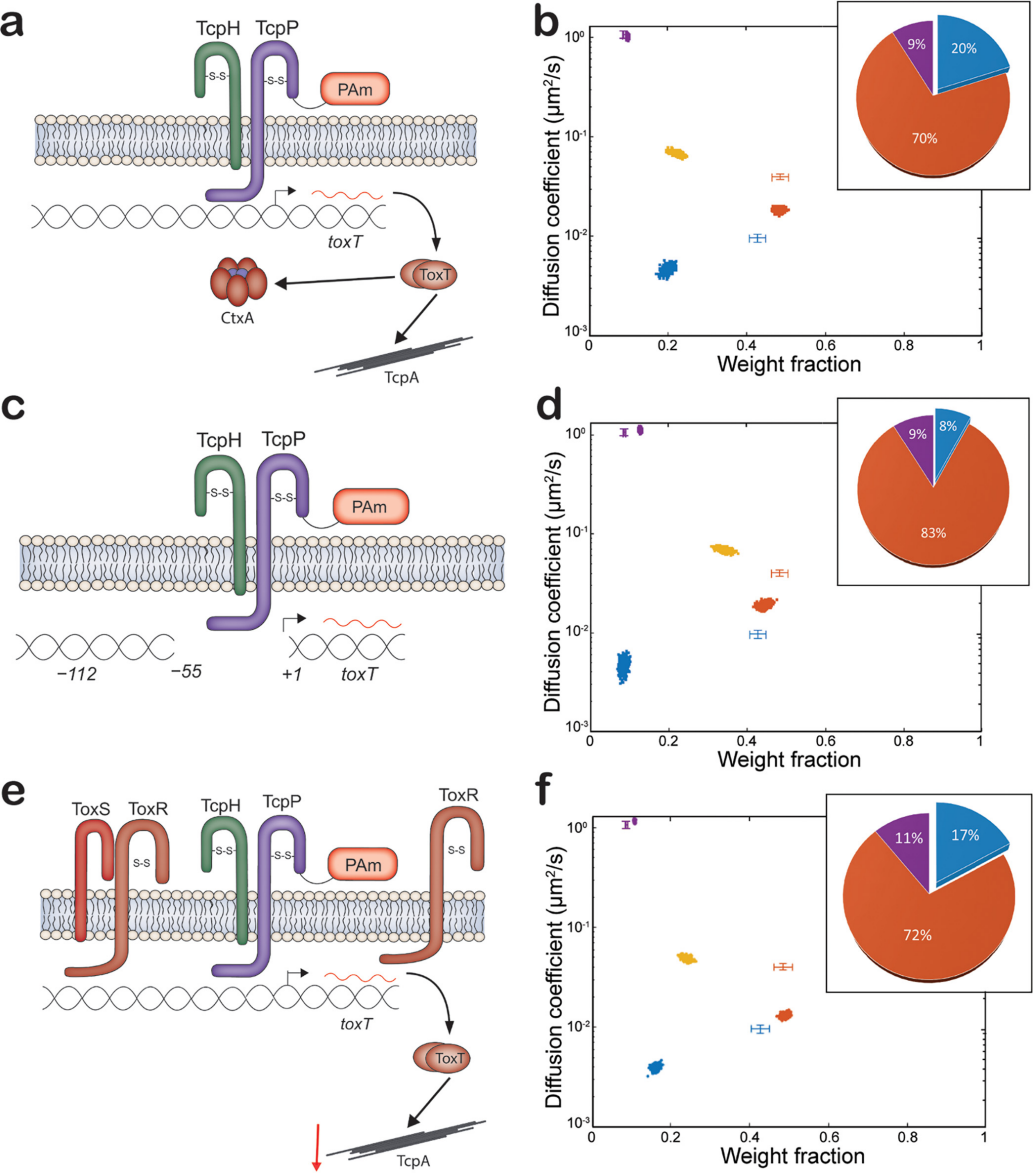
TcpP-PAmCherry diffusion dynamics within live V. cholerae cells lacking ToxRS and regions of the *toxT* promoter. (a, c, and e) Model of *tcpP-PAmCherry* Δ*toxRS*, *tcpP-PAmCherry* Δ*toxRS toxTpro*Δ(−55–+1), and *tcpP-PAmCherry* pMMB66eh-*toxR*, respectively. (b, d, and f) Average single-molecule diffusion coefficients and weight fraction estimates for TcpP-PAmCherry in live V. cholerae *tcpP-PAmCherry* Δ*toxRS* (b), V. cholerae *tcpP-PAmCherry* Δ*toxRS toxTpro*Δ(−55–+1) (d), and *tcpP-PAmCherry* pMMB66eh-*toxR* (f) grown under virulence-inducing conditions. *tcpP-PAmCherry* pMMB66eh-*toxR* was grown in the presence of 1 mM IPTG (isopropyl-β-d-thiogalactopyranoside). Single-step analysis identifies four distinct diffusion states (fast [purple], intermediate [yellow and orange], and slow [blue], respectively). Each point represents the average single-molecule diffusion coefficient versus weight fraction of TcpP-PAmCherry molecules in each distinct mobility state at each saved iteration of the Bayesian algorithm after convergence. The data set contains 80,005 steps from 11,069 trajectories for panel b, 58,577 steps from 11,314 trajectories for panel d, and 134,071 steps from 19,509 trajectories for panel f. The data for TcpP-PAmCherry diffusion in wild-type V. cholerae cells (Fig. 1c) are provided for reference (cross-hairs). (Inset) Percentage (weight fraction) of TcpP-PAmCherry in each diffusion state. Colors as in panel.

**FIG 4:**
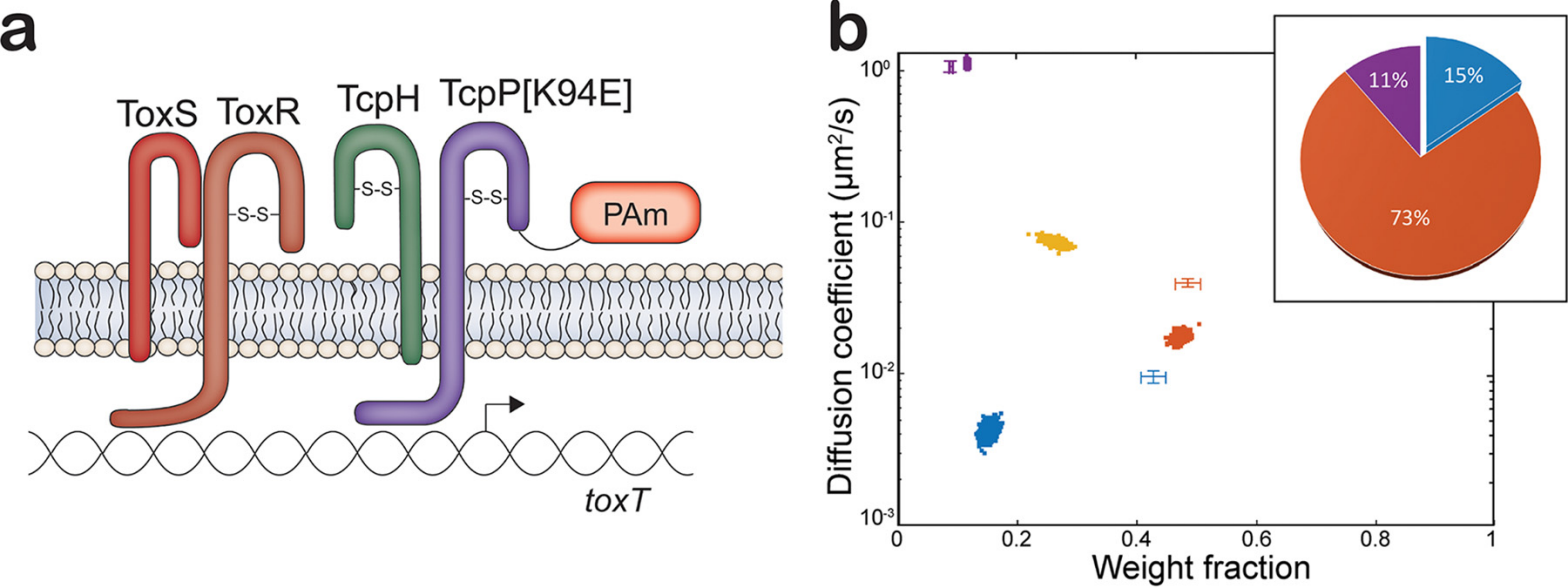
(a) Model of *tcpP-[K94E]-PAmCherry.* (b) Diffusion dynamics of a DNA binding-deficient TcpP-PAmCherry variant within live V. cholerae cells. Average single-molecule diffusion coefficients and weight fraction estimates for TcpP-[K94E]-PAmCherry in live V. cholerae *tcpP-[K94E]-PAmCherry* grown under virulence-inducing conditions. Single-step analysis identifies four distinct diffusion states (fast [purple], intermediate [yellow and orange], and slow [blue], respectively). Each point represents the average single-molecule diffusion coefficient versus weight fraction of TcpP-[K94E]-PAmCherry molecules in each distinct mobility state at each saved iteration of the Bayesian algorithm after convergence. The data set contains 52,565 steps from 8,056 trajectories. The data for TcpP-PAmCherry diffusion in wild-type V. cholerae cells (Fig. 1c) are provided for reference (cross-hairs). (Inset) Percentage (weight fraction) of TcpP-[K94E]-PAmCherry in each diffusion state. Colors as in panel.

Furthermore, our single-step analysis of TcpP-PAmCherry in the *toxTpro*Δ(−112–+1) cells indicates five distinct TcpP-PAmCherry diffusion states, an increase from three states in the wild type (Fig. 2b). In particular, the percentage of TcpP-PAmCherry molecules within the intermediate state overall increased (48% to 78%), but our analysis showed that these moderate moving molecules in fact cluster into three distinct substates (yellow, light orange, and orange in Fig. 2b). These intermediate TcpP-PAmCherry diffusion substates appear when TcpP-PAmCherry is unable to associate with the *toxTpro*. Though large-scale changes in the chromosome structure following the promoter deletion may play a role, these intermediate TcpP-PAmCherry diffusion substates may represent true biochemical interactions that are too short-lived to precisely distinguish and identify due to our current time resolution of 40 ms/acquisition. Further investigation is required to understand the specific biological roles of these substates, but indeed as discussed below, we detect these intermediate substates in all the other mutants studied here (Fig. 3 and 4).

### ToxR promotes TcpP-PAmCherry association with the slow and fast diffusion states.

ToxR is a critical regulator of *toxT* expression through its role supporting TcpP interaction with the *toxTpro* (18, 24, 25). Prior studies have shown that TcpP and ToxR interact in response to low oxygen concentrations, and ToxR antagonizes H-NS from the *toxTpro* (24, 60, 61). Several models for TcpP-mediated *toxT* transcription implicate ToxR in recruitment of TcpP molecules to the *toxTpro* (18, 23–25, 28, 30). Another model invokes “promoter alteration” to suggest that ToxR promotes TcpP-*toxTpro* interaction by displacing the histone-like protein (H-NS) and altering DNA topology rather than recruiting TcpP molecules to the *toxTpro* (28).

To examine the role of ToxR in the motion and localization of TcpP-PAmCherry, we deleted *toxR*, as well as *toxS*, the gene encoding the ToxR accessory protein, in both the *tcpP-PAmCherry* and the *tcpP-PAmCherry toxTpro*Δ(−55–+1) backgrounds, resulting in *tcpP-PAmCherry* Δ*toxRS* and *tcpP-PAmCherry* Δ*toxRS toxTpro*Δ(−55–+1) genotypes. We found that *tcpP-PAmCherry ΔtoxRS* and *tcpP-PAmCherry ΔtoxRS toxTpro*Δ(−55–+1) cells could activate *toxT* transcription, but only *tcpP-PAmCherry ΔtoxRS* supported virulence factor production (Fig. S2A and B and Fig. S4). Complementation of *tcpP-PAmCherry ΔtoxRS* with *toxR* did not change overall levels of TcpA (Fig. S7). Complementation of *tcpP-PAmCherry ΔtoxRS toxTpro*Δ(−55–+1) with ToxR did not restore TcpA to wild-type (WT) levels (Fig. S7). These data show that TcpP-PAmCherry can stimulate *toxT* expression and bind to the *toxTpro* independent of ToxR. WT TcpP can stimulate *toxT* expression independent of ToxR, but only upon TcpP overexpression (18, 24). Due to reduced sensitivity of TcpP-PAmCherry to RIP, we measure higher levels of TcpP-PAmCherry relative to TcpP (Fig. S2A). This observation suggests that cooperativity between ToxR and TcpP is necessary only when levels of TcpP are low (i.e., when TcpP is sensitive to RIP).

10.1128/mBio.02213-21.7 FIG S7 Complementation and overexpression of ToxR from the pMMB66eh plasmid. Western blot assays of cellular lysates collected after growth under virulence-inducing conditions for 6 h with or without IPTG; see Materials and Methods for primary antibody dilution. ToxR does not stimulate TcpA production without TcpPH, and ToxR cannot complement TcpPK94E-PAmCherry or *toxTproΔ*(−55–+1). Low levels of ToxR were detected in *tcpP-PAmCherry ΔtoxRS* and *tcpP-PAmCherry ΔtoxRS toxTpro*Δ(−55–+1) without IPTG, likely due to leaky expression of *toxRS* at the IPTG promoter. Multiple copies of the lac promoter are known to result in leaky expression due to insufficient levels of LacI (62, 63). Download FIG S7, TIF file, 0.07 MB. Copyright © 2021 Calkins et al. 2021 Calkins et al. https://creativecommons.org/licenses/by/4.0/ This content is distributed under the terms of the Creative Commons Attribution 4.0 International license.

The percentage of slowly diffusing TcpP-PAmCherry molecules depends on *toxRS*, as deleting *toxRS* reduces this population in *tcpP-PAmCherry ΔtoxRS* from 43% to 20% (Fig. 3b). This *toxRS* dependence is maintained even in the absence of the TcpP binding site within the *toxT* promoter; the slow population in *tcpP-PAmCherry ΔtoxRS toxTproΔ*(−55–+1) is reduced to 8% from 34% in *tcpP-PAmCherry toxTpro*Δ(−55–+1) (Fig. 3d). Indeed, the TcpP-PAmCherry dynamics are very similar for *tcpP-PAmCherry toxTpro*Δ(−112–+1) (Fig. 2b) and *tcpP-PAmCherry ΔtoxRS toxTpro*Δ(−55–+1) (Fig. 3d). The major difference between TcpP-PAmCherry diffusion dynamics is the loss of the light orange intermediate diffusion substate in *tcpP-PAmCherry ΔtoxRS toxTpro*Δ(−55–+1) (Fig. 3d). These data indicate that, in addition to the slow diffusion state, the presence of ToxR is critical for TcpP-PAmCherry molecules to exist in one of the intermediate substate diffusion states (i.e., the light orange diffusion state).

As shown in Fig. 1d, we found that TcpP-PAmCherry molecules do not freely transition between all the diffusion states: the intermediate diffusion state is an important diffusion state for TcpP-PAmCherry molecules to transition between the fast and the slow diffusion states. Since the ToxR-TcpP interaction is proposed to enable TcpP to associate with the transcription complex at *toxTpro* (18, 24), we reasoned that ToxR is responsible for the preferred intermediate-to-slow-state transition of TcpP-PAmCherry. However, in the Δ*toxRS* mutant (Fig. 3b) as in the wild type (Fig. 1c), only TcpP-PAmCherry molecules in the slowest of the intermediate diffusion substates were likely to transition to the slow diffusion state (orange and blue diffusion states, respectively; Fig. S8b). These transition probabilities suggest that ToxR is not responsible for the restricted transition of TcpP-PAmCherry between the slow and fast diffusion states. Furthermore, the absence of ToxR reduced the probability of TcpP-PAmCherry entering the fast diffusion state and increased the probability of TcpP-PAmCherry leaving the fast diffusion state (Fig. 1d and Fig. S8b). Taken together, these data indicated that ToxR sequesters a portion of the total TcpP-PAmCherry population away from the *toxTpro*. We reasoned that increased levels of ToxR might sequester TcpP molecules to an inactive state (represented by the intermediate diffusion state). To test this hypothesis, we overexpressed ToxR in a *tcpP-PAmCherry* background and quantified virulence factor expression (i.e., TcpA) (Fig. S9). We found that elevated ToxR levels reduced virulence factor levels in both WT and *tcpP-PAmCherry* cells. Furthermore, overexpression of ToxR also decreased the percentage of TcpP-PAmCherry in the slow diffusion state (17% versus 43%) and resulted in the formation of a subintermediate diffusion state, similar to *tcpP-PAmCherry ΔtoxRS* (Fig. 4b). These data suggest that elevated levels of ToxR can repress *toxT* expression by reducing the percentage of TcpP molecules entering the slow diffusion state.

10.1128/mBio.02213-21.8 FIG S8 TcpP-PAmCherry transition plots. Based on the identification of distinct diffusion states for TcpP-PAmCherry (circles with colors as in Fig. 1c and with average single-molecule diffusion coefficient, *D*, indicated in μm^2^/s), the average probabilities of transitioning between mobility states at each step are indicated as arrows between those two circles, and the circle areas are proportional to the weight fractions. Low-significance transition probabilities less than 4% are not displayed. Numbers above the arrows indicate the probability of transition. (a) V. cholerae *tcpP-PAmCherry toxTpro*Δ(−55–+1), corresponding to Fig. 2d. (b) V. cholerae *tcpP-PAmCherry* Δ*toxRS*, corresponding to Fig. 3b. (c) V. cholerae *tcpP-PAmCherry* Δ*toxRS toxTpro*Δ(−55–+1), corresponding to Fig. 3d. (d) V. cholerae *tcpP-PAmCherry pMMB66eh-toxR*, corresponding to Fig. 3f. (e) V. cholerae *tcpP-K94E-PAmCherry*, corresponding to Fig. 4b. Download FIG S8, TIF file, 0.3 MB. Copyright © 2021 Calkins et al. 2021 Calkins et al. https://creativecommons.org/licenses/by/4.0/ This content is distributed under the terms of the Creative Commons Attribution 4.0 International license.

10.1128/mBio.02213-21.9 FIG S9 ToxR overexpression reduces virulence factor production. (A) Western blot assays of cell lysates; three biological replicates, collected after 6 h of virulence-inducing conditions with or without IPTG. (B) Densitometry analysis of the TcpA Western blot in panel A. ImageJ was used to perform the densitometry analysis. Black bars, without IPTG; gray bars, with IPTG. Error bars represent standard deviation. One-tailed Student’s *t* test was used to determine statistical significance. * indicates a *P* value of 0.029. Download FIG S9, TIF file, 0.1 MB. Copyright © 2021 Calkins et al. 2021 Calkins et al. https://creativecommons.org/licenses/by/4.0/ This content is distributed under the terms of the Creative Commons Attribution 4.0 International license.

### Mutation of the TcpP helix-turn-helix domain reduces the percentage of slowly diffusing TcpP-PAmCherry.

Based on results shown in Fig. 1c, we proposed that TcpP-PAmCherry molecules in the slow diffusion state are bound to *toxTpro*, and we found that removing the *toxTpro* binding sites (Fig. 2) or eliminating *toxR* (Fig. 3) significantly reduces this bound state population. Previous studies demonstrated that TcpP does not require DNA binding capability to activate *toxT* expression if ToxR is present (18, 24). To examine this finding further by SMT, we used a *tcpP-PAMCherry* allele with a mutation (K94E) that inhibits TcpP from binding to the *toxTpro* (24). This mutation results in greatly reduced *toxT expression* and TcpA levels (Fig. S2A and Fig. S4). The levels of TcpP[K94E]-PAmCherry are elevated compared with TcpP-PAmCherry (Fig. S2A), consistent with earlier evidence that the K94E substitution increases TcpP stability (24). In addition to TcpP[K94E]-PAmCherry being unable to stimulate *toxT* expression, a lower percentage of TcpP[K94E]-PAmCherry molecules are detected in the slowest-diffusing state than for TcpP-PAmCherry (15% versus 43%; Fig. 4b). Furthermore, TcpP[K94E]-PAmCherry molecules have an additional intermediate diffusion substate, similar to both *tcpP-PAmCherry ΔtoxRS* and *tcpP-PAmCherry ΔtoxRS toxTproΔ*(−55–+1) (Fig. 4b).

## DISCUSSION

How MLTAs find their target sequences from the membrane represents a major gap in knowledge. Here, we started to address this by investigating single-molecule dynamics of TcpP-PAmCherry. Taken together with previous work, the data presented here demonstrate that TcpP-PAmCherry molecules diffuse in at least three distinct biophysical states (fast, intermediate, and slow diffusion) but do not freely transition between all diffusion states (54). We hypothesized that each of these biochemical states have distinct biological roles. Specifically, we hypothesized that the slow diffusion state represented TcpP-PAmCherry molecules interacting with the *toxTpro*. To test this hypothesis, we made targeted deletions to the *toxTpro* and of *toxRS*, and we mutated the TcpP DNA binding domain (K94E). Our biophysical measurements of these mutations support the hypothesis that the slow diffusion state is occupied by TcpP-PAmCherry molecules interacting specifically with DNA at *toxTpro*. Additionally, we observed that TcpP-PAmCherry molecules only transition to the slow diffusion state from the intermediate diffusion state and that ToxR is not responsible for this transition specificity. These data support a modified promoter alteration model (28) in which ToxR binds to the distal region of the *toxTpro* to promote TcpP binding to the proximal region of the *toxTpro* or, in the absence of its binding site, ToxR directly interacts with TcpP to stimulate *toxT* expression. Our data do not suggest that ToxR directs or recruits TcpP to the *toxTpro*.

While ToxR is critical for TcpP to stimulate *toxT* expression (18, 24, 27), our data demonstrate that TcpP-PAmCherry can support *toxT* expression and virulence factor production without ToxR, which may be a consequence of the greater stability of TcpP-PAmCherry than of native TcpP (see Fig. S2A and Fig. S4 in the supplemental material). Moreover, our single-molecule imaging finds a higher percentage of the TcpP-PAmCherry molecules in the slow diffusion state in *tcpP-PAmCherry ΔtoxRS* cells than in *tcpP-PAmCherry ΔtoxRS toxTpro*Δ(−55–+1) cells (Fig. 3). In addition, prior DNase I footprinting experiments have demonstrated that in cells lacking *toxR* TcpP protects a larger region of the *toxTpro* (−100 to −32), i.e., TcpP protects most of the ToxR binding and TcpP binding sites in the *ΔtoxRS* mutant (18). Taken together, these results indicate that (i) ToxR is not essential for TcpP to locate the *toxTpro* and (ii) TcpP is able to interact with the *toxTpro* independent of ToxR. In addition, our data show that Δ*toxRS* reduces the percentage of DNA-bound TcpP-PAmCherry but does not decrease the probability of TcpP-PAmCherry molecules transitioning from the intermediate state to the bound state (Fig. 3 and Fig. S8b). Despite a reduction in the percentage of DNA-bound TcpP-PAmCherry, TcpP-PAmCherry stimulates WT *toxT* expression independent of ToxR (Fig. S4). These data support the promoter alteration model (28) in which, rather than ToxR recruiting TcpP to the *toxTpro*, ToxR assists TcpP to stimulate *toxT* transcription once TcpP independently associates with the *toxTpro*. Counterintuitively, in the absence of ToxRS TcpP-PAmCherry molecules have a lower probability of exiting the slow diffusion state (Fig. S8b). Given that RIP of TcpP-PAmCherry impedes our ability to image TcpP-PAmCherry, these data suggest that TcpP-PAmCherry molecules might be sensitive to RIP while interacting with the *toxTpro* and that ToxRS may inhibit RIP of TcpP while interacting with the *toxTpro*. If this is the case, given that we are unable to image TcpP-PAmCherry molecules that are sensitive to RIP, it might explain why we observe a lower percentage of TcpP-PAmCherry molecules in the slow diffusion state and yet we observe WT *toxT* expression in the absence of ToxRS. However, future experiments are required to determine if ToxRS inhibits RIP of TcpP while interacting with the *toxTpro*.

Under certain conditions, ToxR can negatively influence *toxT* expression. In response to stationary-phase accumulation of the cyclic dipeptide cyclic phenylalanine-proline (cyc-Phe-Pro), ToxR stimulates production of LeuO, resulting in downregulation of the *tcpP* regulator *aphA* (62, 63). Our data suggest that ToxR can also reduce *toxT* expression by influencing TcpP-PAmCherry single-molecule dynamics (Fig. S8b). Deletion of *toxRS* reduces the overall probability of TcpP-PAmCherry molecules transitioning between the intermediate and fast diffusion states (Fig. S8b). Moreover, elevated levels of ToxR reduce both the percentage of TcpP-PAmCherry in the slow diffusion state and virulence factor production (Fig. 3f and Fig. S9), suggesting that ToxR can antagonize *toxT* expression by promoting transition of TcpP molecules to the fast or subintermediate diffusion states. A similar phenotype has been reported previously (18). Lastly, prior electrophoretic mobility shift assays also indicate that ToxR can sequester TcpP from the *toxTpro*. In *ΔtoxRS* cells TcpP is able to bind to the *toxTpro* −73–+45 (*toxTpro* lacking the ToxR binding region), but not in the presence of ToxR molecules (18). It remains unclear how ToxR sequesters TcpP-PAmCherry molecules from the slow diffusion state. However, we hypothesize that ToxR promotes TcpP molecules to transition away from the slow diffusion state to prevent aberrant *toxT* expression. Follow-up experiments are required to test this hypothesis.

Currently, the biological roles of the intermediate diffusion states (or intermediate diffusion substates) are unclear, but the intermediate states are certainly important, as TcpP molecules transition to the *toxTpro*-bound state from them. There is nearly a 10-fold difference in diffusion coefficients between the slow and intermediate diffusion states (0.006 μm^2^/s versus 0.044 μm^2^/s, respectively; Fig. 1c). This difference cannot be explained by dimerization or interaction of ToxR and TcpP-PAmCherry alone: the mobility of membrane-localized proteins scales linearly with the number of transmembrane helices, such that increasing the number of transmembrane helices via dimerization from one to two would reduce the diffusion coefficient only by a factor of two (64). One possibility is that TcpP-PAmCherry molecules undergo fast diffusion in less protein-dense areas of the cytoplasmic membrane relative to TcpP-PAmCherry molecules undergoing intermediate diffusion. Prior single-molecule analysis of 209 membrane-localized proteins in Bacillus subtilis revealed that only 6% of all membrane proteins imaged were homogeneously distributed throughout the cytoplasmic membrane (65). Heterogeneous distribution of membrane-localized proteins in B. subtilis suggests that similar distribution of membrane-localized proteins in V. cholerae can occur. It remains unclear why the vast majority of these membrane-localized proteins in B. subtilis have heterogeneous diffusion dynamics. One possibility is that these membrane-localized proteins have different preferences for lipid-ordered and lipid-disordered membrane domains. Prior studies have demonstrated that transmembrane domain properties (e.g., surface area, length, and posttranslational modifications) are major factors in determining lipid-ordered or lipid-disordered membrane domain preference (66). We are currently exploring if lipid-ordered and lipid-disordered membrane domains influence diffusion dynamics of TcpP molecules within the fast and intermediate diffusion states.

Alternatively, it is possible that the diffusion coefficients of TcpP-PAmCherry molecules in the intermediate state are undergoing nonspecific interactions with DNA whereas the slowest TcpP-PAmCherry molecules are specifically bound at *toxTpro*. Our data show that there are some slow-moving TcpP-PAmCherry molecules when major regions of the *toxTpro* are deleted or when key residues within the DNA binding domain of TcpP are mutated (i.e., *tcpP[K94E]-PAmCherry*; Fig. 2 and 4). When considering our alternative model of nonspecific DNA binding by TcpP, our data suggest two possibilities: (i) TcpP-PAmCherry molecules in the slow diffusion state represent TcpP molecules that make both specific and nonspecific interactions with DNA or (ii) TcpP-PAmCherry molecules in the slow diffusion state interact specifically with non-*toxTpro* DNA (i.e., TcpP regulates additional genes). Several genes appear to have altered gene expression upon deletion of *tcpPH* (67). However, these experiments have yet to be replicated. Thus, future experiments would be required to test these hypotheses.

These results provide deep insights that further expand the model of cooperativity between ToxR and TcpP-PAmCherry. Our data demonstrate that ToxR assists TcpP to associate with the *toxTpro* even in the absence of the TcpP binding site, further supporting the established model of cooperativity between TcpP and ToxR. The data also show that TcpP can locate the *toxTpro*, interact with the *toxTpro*, and stimulate *toxT* expression independent of ToxR. This supports the promoter alteration model in which TcpP molecules independently associate with the *toxTpro* while ToxR enhances this association by altering *toxTpro* topology to stimulate *toxT* transcription. In addition to independently associating with the *toxTpro*, these data show that ToxR promotes transition of TcpP molecules to the fast and subintermediate diffusion states, shifting the equilibrium of TcpP molecules away from the *toxTpro*. The mechanism by which ToxR promotes transition of TcpP molecules away from the slow diffusion state is currently unclear but will be the subject of future investigation. Given that *toxT* expression is highly regulated, we speculate that sequestration of TcpP molecules from the *toxTpro* is yet another mechanism to fine-tune *toxT* expression. It is probable that other MLTAs, found in both Gram-negative and Gram-positive bacteria, have similar biophysical properties (Fig. S1). Continued exploration of MLTA biophysical properties could be leveraged to develop alternative strategies to inhibit MLTAs to treat bacterial infections without exacerbating the global antibiotic resistance crisis.

## MATERIALS AND METHODS

### Bacterial strains and growth conditions.

Escherichia coli and V. cholerae strains used here can be found in Table S1A in the supplemental material. Unless otherwise stated, E. coli and V. cholerae cells were grown on lysogeny broth (LB) plates, or in LB at 210 rpm, at 37°C. LB was prepared according to previous descriptions (68). To stimulate virulence, V. cholerae cells were diluted from overnight cultures in LB and subcultured under virulence-inducing conditions: LB, pH 6.5, 110 rpm, 30°C; filter sterilized. Here, the LB pH was adjusted by adding HCl (1 N) to pH 6.5 (±0.05), and then the medium was filter sterilized to maintain pH. Where appropriate, antibiotics and cell wall intermediates were added at the concentrations given in parentheses: streptomycin (100 μg ml^−1^), ampicillin (100 μg ml^−1^), and diaminopimelic acid (DAP) (300 μM).

10.1128/mBio.02213-21.10 TABLE S1 (A) Strain list. (B) Primer list. Kpn1-HiFi restriction sites were included in forward primers, and Xba1 restriction sites were included in all reverse primers to provide homology between insert and vector sequences. Download Table S1, DOCX file, 0.01 MB. Copyright © 2021 Calkins et al. 2021 Calkins et al. https://creativecommons.org/licenses/by/4.0/ This content is distributed under the terms of the Creative Commons Attribution 4.0 International license.

### Plasmid construction.

Plasmid vectors were purified using the Qiagen miniprep kit. Plasmid inserts were amplified from V. cholerae genomic DNA using Phusion high-fidelity polymerase (Thermo Scientific). Splicing by overlap extension was used to combine the entire plasmid insert sequences together; see Table S1B for the primer list. Plasmid vector was digested by restriction digestion using KpnI-HiFi and XbaI (New England BioLabs) at 37°C for 2 h. After digestion, the plasmid vector and insert were added to Gibson assembly master mix (1.5 μl insert, 0.5 μl vector, 2 μl master mix) (New England BioLabs) and incubated at 50°C for 1 h. Assembled plasmid was electroporated into E. coli λpir cells and recovered on LB plates with ampicillin and DAP.

### Bacterial strain construction.

Strain construction follows the protocol outlined in reference 69. Briefly, E. coli λpir harboring the pKAS plasmid and the donor V. cholerae strain were incubated in LB (broth or agar) supplemented with DAP overnight at 37°C. The remaining cells were then spread on LB plates containing ampicillin or thiosulfate-citrate-bile-sucrose (TCBS) plates containing ampicillin. Counterselection for loss of the pKAS construct by V. cholerae cells was done by incubating cells in LB for 2 h and then for 2 h with 2,500 μg ml^−1^ streptomycin (both at 37°C, 210 rpm). Twenty microliters of this culture was spread onto LB plates containing 2,500 μg ml^−1^ of streptomycin and incubated overnight at 37°C. Streptomycin-resistant colonies were screened for the chromosomal mutation of interest via colony PCR using *Taq* DNA polymerase (Thermo Fisher). Genomic DNA was purified from possible mutants and sequenced (Genewiz) to validate the exchange. Because overlapping open reading frames encode TcpP and TcpH, *tcpH* was cloned downstream of PAmCherry to maintain its expression, and a stop codon was introduced within the first three codons of the native *tcpH* coding sequence to prevent out-of-frame translation of PAmCherry.

### Growth curves.

V. cholerae strains were initially grown on LB plates containing streptomycin (100 μg ml^−1^) overnight at 37°C, and then an individual colony was picked and grown overnight in LB at 37°C. V. cholerae cells were diluted to an optical density at 600 nm (OD_600_) of 0.01 from the overnight LB into a 96-well plate (Cell Pro) with 200 μl of virulence-inducing medium per well. The plate was then incubated at 30°C with shaking every 30 min before each measurement in a SPECTROstar Omega plate reader (BMG Labtech).

### RT-qPCR.

RNA was extracted from V. cholerae cells grown under virulence-inducing conditions. RNA was preserved by resuspending pellet cells in 1 ml TRIzol (Sigma-Aldrich) and then purified using an RNeasy kit (Qiagen). RNA was further purified with Turbo DNase treatment. RNA quantity and quality were measured via UV-visible (UV-Vis) spectrophotometry (NanoDrop ND-1000) and by detection of large and small ribosomal subunits via 2% agarose gel. RNA was then converted to cDNA using Superscript III reverse transcriptase (Thermo Scientific). Real-time quantitative PCR (RT-qPCR) was performed using 5 ng of cDNA in SYBR green master mix (Applied Biosystems). RecA was used as a housekeeping gene of reference to calculate the cycle threshold values (ΔΔ*C_T_*) (70, 74). See Table S1B for primers.

### Protein electrophoresis and immunodetection.

After lysis, total protein concentration samples were measured via Bradford assay. Samples were subsequently diluted to 0.5 μg total protein/μl. All SDS-PAGE gels contained 12.5% acrylamide and were run at 90 to 120 V for 1.5 h. Proteins were transferred to nitrocellulose membranes using a semidry electroblotter (Fisher Scientific) overnight at 35 mA or for 2 h at 200 mA. Membranes were blocked with 5% nonfat milk, 2% bovine serum albumin in Tris-buffered saline, 0.5% Tween 20 (TBST) for 1 h. Membranes were then incubated with primary antibody (anti-TcpA, 1:100,000; anti-TcpP, 1:1,000; anti-TcpH, 1:500; anti-ToxR, 1:50,000; anti-mCherry, 1:1,000) diluted in TBST and nonfat milk (2.5%, wt/vol) for an additional hour at room temperature with shaking. Membranes were then washed 3 times with TBST. Secondary antibody (goat anti-rabbit IgG-horseradish peroxidase [HRP], 1:2,000) (Sigma) was diluted in TBST and nonfat milk (2.5%, wt/vol). Secondary antibody was incubated with the membranes for an additional hour at room temperature with shaking. Membranes were washed again with TBST 3 times and then incubated with SuperSignal HRP chemiluminescence substrate (Thermo Fisher). Membranes were imaged with an Amersham Imager 600.

### Single-molecule microscopy.

V. cholerae strains were grown on LB plates containing streptomycin (100 μg ml^−1^) overnight at 37°C, and then an individual colony was picked and grown overnight in LB at 37°C. V. cholerae cells were diluted from LB under virulence-inducing conditions and grown until they reached mid-log phase. They were then washed and concentrated in M9 minimal medium with 0.4% glycerol. A 1.5-μl droplet of concentrated cells was placed onto an agarose pad (2% agarose in M9, spread and flattened on a microscope slide) and covered with a coverslip. Cells were imaged at room temperature using an Olympus IX71 inverted epifluorescence microscope with a 100× 1.40-numerical-aperture (NA) oil-immersion objective, a 405-nm laser (Coherent Cube 405-100; 50 W/cm^2^) for photoactivation, and a coaligned 561-nm laser (Coherent-Sapphire 561-50; 210 W/cm^2^) for fluorescence excitation. Fluorescence emission was filtered with appropriate filters and captured on a 512- by 512-pixel Photometrics Evolve electron-multiplying charge-coupled device (EMCCD) camera. To prevent higher-order excitation during photoactivation, a pair of Uniblitz shutters controlled the laser beams such that samples were exposed to only one laser at a time. During imaging, the cells were given a 40-ms dose of 405-nm light every 90 s. Images were collected continuously every 40 ms, and acquisitions lasted 5 to 7 min each.

### Data analysis.

Recorded single-molecule positions were detected and localized based on point spread function fitting using home-built code, SMALL-LABS (71). This program reduces biases due to background subtraction, increasing the precision of each molecule localization. Subsequent localizations of the same molecule were then connected into trajectories using the Hungarian algorithm (72, 73). All trajectories from each movie for a given condition were combined and analyzed together using the Single-Molecule Analysis by Unsupervised Gibbs sampling (SMAUG) algorithm (54). This algorithm considers the collection of steps in all trajectories and uses a Bayesian statistical framework to estimate the parameters of interest: number of mobility states, diffusion coefficient, weight fraction, transition probabilities between states, and noise.

### Data availability.

The data presented here will be made available from the corresponding authors upon request.

## References

[1] Zuckerman JN, Rombo L, Fisch A. 2007. The true burden and risk of cholera: implications for prevention and control. Lancet Infect Dis 7:521–530. doi:10.1016/S1473-3099(07)70138-X. 17584531

[2] Baker-Austin C, Oliver JD, Alam M, Ali A, Waldor MK, Qadri F, Martinez-Urtaza J. 2018. Vibrio spp. infections. Nat Rev Dis Primers 4:8. doi:10.1038/s41572-018-0005-8. 30002421

[3] Jelinek T, Kollaritsch H. 2008. Vaccination with Dukoral against travelers’ diarrhea (ETEC) and cholera. Expert Rev Vaccines 7:561–567. doi:10.1586/14760584.7.5.561. 18564011

[4] Saha A, Chowdhury MI, Khanam F, Bhuiyan MS, Chowdhury F, Khan AI, Khan IA, Clemens J, Ali M, Cravioto A, Qadri F. 2011. Safety and immunogenicity study of a killed bivalent (O1 and O139) whole-cell oral cholera vaccine Shanchol, in Bangladeshi adults and children as young as 1 year of age. Vaccine 29:8285–8292. doi:10.1016/j.vaccine.2011.08.108. 21907255

[5] Cabrera A, Lepage JE, Sullivan KM, Seed SM. 2017. Vaxchora: a single-dose oral cholera vaccine. Ann Pharmacother 51:584–589. doi:10.1177/1060028017698162. 28622736

[6] Kim HB, Wang M, Ahmed S, Park CH, LaRocque RC, Faruque ASG, Salam MA, Khan WA, Qadri F, Calderwood SB, Jacoby GA, Hooper DC. 2010. Transferable quinolone resistance in Vibrio cholerae. Antimicrob Agents Chemother 54:799–803. doi:10.1128/AAC.01045-09. 19949057 PMC2812163

[7] Krishna BVS, Patil AB, Chandrasekhar MR. 2006. Fluoroquinolone-resistant Vibrio cholerae isolated during a cholera outbreak in India. Trans R Soc Trop Med Hyg 100:224–226. doi:10.1016/j.trstmh.2005.07.007. 16246383

[8] Glass RI, Huq MI, Lee JV, Threlfall EJ, Khan MR, Alim AR, Rowe B, Gross RJ. 1983. Plasmid-borne multiple drug resistance in Vibrio cholerae serogroup O1, biotype El Tor: evidence for a point-source outbreak in Bangladesh. J Infect Dis 147:204–209. doi:10.1093/infdis/147.2.204. 6827137

[9] Pang B, Du P, Zhou Z, Diao B, Cui Z, Zhou H, Kan B. 2016. The transmission and antibiotic resistance variation in a multiple drug resistance clade of Vibrio cholerae circulating in multiple countries in Asia. PLoS One 11:e0149742. doi:10.1371/journal.pone.0149742. 26930352 PMC4773069

[10] Ramakrishna BS, Venkataraman S, Srinivasan P, Dash P, Young GP, Binder HJ. 2000. Amylase-resistant starch plus oral rehydration solution for cholera. N Engl J Med 342:308–313. doi:10.1056/NEJM200002033420502. 10655529

[11] Chowdhury FR, Nur Z, Hassan N, von Seidlein L, Dunachie S. 2017. Pathogenicity and changing molecular epidemiology of cholera in the era of global warming. Ann Clin Microbiol Antimicrob 16:10. doi:10.1186/s12941-017-0185-1. 28270154 PMC5341193

[12] Angelichio MJ, Spector J, Waldor MK, Camilli A. 1999. Vibrio cholerae intestinal population dynamics in the suckling mouse model of infection. Infect Immun 67:3733–3739. doi:10.1128/IAI.67.8.3733-3739.1999. 10417131 PMC96647

[13] Millet YA, Alvarez D, Ringgaard S, von Andrian UH, Davis BM, Waldor MK. 2014. Insights into Vibrio cholerae intestinal colonization from monitoring fluorescently labeled bacteria. PLoS Pathog 10:e1004405. doi:10.1371/journal.ppat.1004405. 25275396 PMC4183697

[14] Taylor RK, Miller VL, Furlong DB, Mekalanos JJ. 1987. Use of phoA gene fusions to identify a pilus colonization factor coordinately regulated with cholera toxin. Proc Natl Acad Sci USA 84:2833–2837. doi:10.1073/pnas.84.9.2833. 2883655 PMC304754

[15] Nelson EJ, Harris JB, Morris JG, Jr, Calderwood SB, Camilli A. 2009. Cholera transmission: the host, pathogen and bacteriophage dynamic. Nat Rev Microbiol 7:693–702. doi:10.1038/nrmicro2204. 19756008 PMC3842031

[16] Camilli A, Beattie DT, Mekalanos JJ. 1994. Use of genetic recombination as a reporter of gene expression. Proc Natl Acad Sci USA 91:2634–2638. doi:10.1073/pnas.91.7.2634. 8146167 PMC43424

[17] Nielsen AT, Dolganov NA, Rasmussen T, Otto G, Miller MC, Felt SA, Torreilles S, Schoolnik GK. 2010. A bistable switch and anatomical site control Vibrio cholerae virulence gene expression in the intestine. PLoS Pathog 6:e1001102. doi:10.1371/journal.ppat.1001102. 20862321 PMC2940755

[18] Krukonis ES, Yu RR, Dirita VJ. 2000. The Vibrio cholerae ToxR/TcpP/ToxT virulence cascade: distinct roles for two membrane-localized transcriptional activators on a single promoter. Mol Microbiol 38:67–84. doi:10.1046/j.1365-2958.2000.02111.x. 11029691

[19] DiRita VJ, Parsot C, Jander G, Mekalanos JJ. 1991. Regulatory cascade controls virulence in Vibrio cholerae. Proc Natl Acad Sci USA 88:5403–5407. doi:10.1073/pnas.88.12.5403. 2052618 PMC51881

[20] Higgins DE, DiRita VJ. 1994. Transcriptional control of toxT, a regulatory gene in the ToxR regulon of Vibrio cholerae. Mol Microbiol 14:17–29. doi:10.1111/j.1365-2958.1994.tb01263.x. 7830555

[21] Higgins DE, Nazareno E, DiRita VJ. 1992. The virulence gene activator ToxT from Vibrio cholerae is a member of the AraC family of transcriptional activators. J Bacteriol 174:6974–6980. doi:10.1128/jb.174.21.6974-6980.1992. 1400247 PMC207377

[22] Miller VL, Taylor RK, Mekalanos JJ. 1987. Cholera toxin transcriptional activator ToxR is a transmembrane DNA binding protein. Cell 48:271–279. doi:10.1016/0092-8674(87)90430-2. 3802195

[23] Crawford JA, Krukonis ES, DiRita VJ. 2003. Membrane localization of the ToxR winged-helix domain is required for TcpP-mediated virulence gene activation in Vibrio cholerae. Mol Microbiol 47:1459–1473. doi:10.1046/j.1365-2958.2003.03398.x. 12603748

[24] Krukonis ES, DiRita VJ. 2003. DNA binding and ToxR responsiveness by the wing domain of TcpP, an activator of virulence gene expression in Vibrio cholerae. Mol Cell 12:157–165. doi:10.1016/S1097-2765(03)00222-3. 12887901

[25] Goss TJ, Seaborn CP, Gray MD, Krukonis ES. 2010. Identification of the TcpP-binding site in the toxT promoter of Vibrio cholerae and the role of ToxR in TcpP-mediated activation. Infect Immun 78:4122–4133. doi:10.1128/IAI.00566-10. 20679441 PMC2950353

[26] Carroll PA, Tashima KT, Rogers MB, DiRita VJ, Calderwood SB. 1997. Phase variation in tcpH modulates expression of the ToxR regulon in Vibrio cholerae. Mol Microbiol 25:1099–1111. doi:10.1046/j.1365-2958.1997.5371901.x. 9350866

[27] Häse CC, Mekalanos JJ. 1998. TcpP protein is a positive regulator of virulence gene expression in Vibrio cholerae. Proc Natl Acad Sci USA 95:730–734. doi:10.1073/pnas.95.2.730. 9435261 PMC18489

[28] Goss TJ, Morgan SJ, French EL, Krukonis ES. 2013. ToxR recognizes a direct repeat element in the toxT, ompU, ompT, and ctxA promoters of Vibrio cholerae to regulate transcription. Infect Immun 81:884–895. doi:10.1128/IAI.00889-12. 23297386 PMC3584884

[29] Martínez-Hackert E, Stock AM. 1997. Structural relationships in the OmpR family of winged-helix transcription factors. J Mol Biol 269:301–312. doi:10.1006/jmbi.1997.1065. 9199401

[30] Haas BL, Matson JS, DiRita VJ, Biteen JS. 2015. Single-molecule tracking in live Vibrio cholerae reveals that ToxR recruits the membrane-bound virulence regulator TcpP to the toxT promoter. Mol Microbiol 96:4–13. doi:10.1111/mmi.12834. 25318589 PMC6025817

[31] Ulrich LE, Koonin EV, Zhulin IB. 2005. One-component systems dominate signal transduction in prokaryotes. Trends Microbiol 13:52–56. doi:10.1016/j.tim.2004.12.006. 15680762 PMC2756188

[32] Parkinson JS, Kofoid EC. 1992. Communication modules in bacterial signaling proteins. Annu Rev Genet 26:71–112. doi:10.1146/annurev.ge.26.120192.000443. 1482126

[33] Haas BL, Matson JS, DiRita VJ, Biteen JS. 2014. Imaging live cells at the nanometer-scale with single-molecule microscopy: obstacles and achievements in experiment optimization for microbiology. Molecules 19:12116–12149. doi:10.3390/molecules190812116. 25123183 PMC4346097

[34] Hanson BR, Lowe BA, Neely MN. 2011. Membrane topology and DNA-binding ability of the streptococcal CpsA protein. J Bacteriol 193:411–420. doi:10.1128/JB.01098-10. 21097630 PMC3019820

[35] Cieslewicz MJ, Kasper DL, Wang Y, Wessels MR. 2001. Functional analysis in type Ia group B Streptococcus of a cluster of genes involved in extracellular polysaccharide production by diverse species of streptococci. J Biol Chem 276:139–146. doi:10.1074/jbc.M005702200. 11027683

[36] Gebhard S, Gaballa A, Helmann JD, Cook GM. 2009. Direct stimulus perception and transcription activation by a membrane-bound DNA binding protein. Mol Microbiol 73:482–491. doi:10.1111/j.1365-2958.2009.06787.x. 19602149 PMC2752741

[37] Matos R, Pinto VV, Ruivo M, de Fátima Silva Lopes M. 2009. Study on the dissemination of the bcrABDR cluster in Enterococcus spp. reveals that the BcrAB transporter is sufficient to confer high-level bacitracin resistance. Int J Antimicrob Agents 34:142–147. doi:10.1016/j.ijantimicag.2009.02.008. 19324527

[38] Blanc-Potard AB, Solomon F, Kayser J, Groisman EA. 1999. The SPI-3 pathogenicity island of Salmonella enterica. J Bacteriol 181:998–1004. doi:10.1128/JB.181.3.998-1004.1999. 9922266 PMC93469

[39] Tükel C, Akçelik M, de Jong MF, Simsek O, Tsolis RM, Bäumler AJ. 2007. MarT activates expression of the MisL autotransporter protein of Salmonella enterica serotype Typhimurium. J Bacteriol 189:3922–3926. doi:10.1128/JB.01746-06. 17351045 PMC1913337

[40] Yeo W-S, Anokwute C, Marcadis P, Levitan M, Ahmed M, Bae Y, Kim K, Kostrominova T, Liu Q, Bae T. 2020. A membrane-bound transcription factor is proteolytically regulated by the AAA+ protease FtsH in Staphylococcus aureus. J Bacteriol 202:e00019-20. doi:10.1128/JB.00019-20. 32094161 PMC7148131

[41] Hubbard TP, Chao MC, Abel S, Blondel CJ, Abel Zur Wiesch P, Zhou X, Davis BM, Waldor MK. 2016. Genetic analysis of Vibrio parahaemolyticus intestinal colonization. Proc Natl Acad Sci USA 113:6283–6288. doi:10.1073/pnas.1601718113. 27185914 PMC4896720

[42] Kuper C, Jung K. 2005. CadC-mediated activation of the cadBA promoter in Escherichia coli. J Mol Microbiol Biotechnol 10:26–39. doi:10.1159/000090346. 16491024

[43] Dalia AB, Lazinski DW, Camilli A. 2014. Identification of a membrane-bound transcriptional regulator that links chitin and natural competence in Vibrio cholerae. mBio 5:e01028-13. doi:10.1128/mBio.01028-13. 24473132 PMC3903286

[44] Sobetzko P, Travers A, Muskhelishvili G. 2012. Gene order and chromosome dynamics coordinate spatiotemporal gene expression during the bacterial growth cycle. Proc Natl Acad Sci USA 109:E42–E50. doi:10.1073/pnas.1108229109. 22184251 PMC3258614

[45] Browning DF, Grainger DC, Busby SJ. 2010. Effects of nucleoid-associated proteins on bacterial chromosome structure and gene expression. Curr Opin Microbiol 13:773–780. doi:10.1016/j.mib.2010.09.013. 20951079

[46] Liu LF, Wang JC. 1987. Supercoiling of the DNA template during transcription. Proc Natl Acad Sci USA 84:7024–7027. doi:10.1073/pnas.84.20.7024. 2823250 PMC299221

[47] Harrington EW, Trun NJ. 1997. Unfolding of the bacterial nucleoid both in vivo and in vitro as a result of exposure to camphor. J Bacteriol 179:2435–2439. doi:10.1128/jb.179.7.2435-2439.1997. 9079934 PMC178985

[48] Dorman CJ. 1991. DNA supercoiling and environmental regulation of gene expression in pathogenic bacteria. Infect Immun 59:745–749. doi:10.1128/iai.59.3.745-749.1991. 1997427 PMC258322

[49] Badrinarayanan A, Le TBK, Laub MT. 2015. Bacterial chromosome organization and segregation. Annu Rev Cell Dev Biol 31:171–199. doi:10.1146/annurev-cellbio-100814-125211. 26566111 PMC4706359

[50] Brameyer S, Rösch TC, El Andari J, Hoyer E, Schwarz J, Graumann PL, Jung K. 2019. DNA-binding directs the localization of a membrane-integrated receptor of the ToxR family. Commun Biol 2:4. doi:10.1038/s42003-018-0248-7. 30740540 PMC6320335

[51] Valens M, Penaud S, Rossignol M, Cornet F, Boccard F. 2004. Macrodomain organization of the Escherichia coli chromosome. EMBO J 23:4330–4341. doi:10.1038/sj.emboj.7600434. 15470498 PMC524398

[52] Cagliero C, Grand RS, Jones MB, Jin DJ, O’Sullivan JM. 2013. Genome conformation capture reveals that the Escherichia coli chromosome is organized by replication and transcription. Nucleic Acids Res 41:6058–6071. doi:10.1093/nar/gkt325. 23632166 PMC3695519

[53] Le TBK, Imakaev MV, Mirny LA, Laub MT. 2013. High-resolution mapping of the spatial organization of a bacterial chromosome. Science 342:731–734. doi:10.1126/science.1242059. 24158908 PMC3927313

[54] Karslake JD, Donarski ED, Shelby SA, Demey LM, DiRita VJ, Veatch SL, Biteen JS. 2021. SMAUG: analyzing single-molecule tracks with nonparametric Bayesian statistics. Methods 193:16–26. doi:10.1016/j.ymeth.2020.03.008. 32247784 PMC7529709

[55] Teoh WP, Matson JS, DiRita VJ. 2015. Regulated intramembrane proteolysis of the virulence activator TcpP in Vibrio cholerae is initiated by the tail-specific protease (Tsp). Mol Microbiol 97:822–831. doi:10.1111/mmi.13069. 25999037

[56] Matson JS, DiRita VJ. 2005. Degradation of the membrane-localized virulence activator TcpP by the YaeL protease in Vibrio cholerae. Proc Natl Acad Sci USA 102:16403–16408. doi:10.1073/pnas.0505818102. 16254052 PMC1283431

[57] Beck NA, Krukonis ES, DiRita VJ. 2004. TcpH influences virulence gene expression in Vibrio cholerae by inhibiting degradation of the transcription activator TcpP. J Bacteriol 186:8309–8316. doi:10.1128/JB.186.24.8309-8316.2004. 15576780 PMC532408

[58] Hay AJ, Yang M, Xia X, Liu Z, Hammons J, Fenical W, Zhu J. 2017. Calcium enhances bile salt-dependent virulence activation in Vibrio cholerae. Infect Immun 85:e00707-16. doi:10.1128/IAI.00707-16. 27849180 PMC5203667

[59] Yang M, Liu Z, Hughes C, Stern AM, Wang H, Zhong Z, Kan B, Fenical W, Zhu J. 2013. Bile salt-induced intermolecular disulfide bond formation activates Vibrio cholerae virulence. Proc Natl Acad Sci USA 110:2348–2353. doi:10.1073/pnas.1218039110. 23341592 PMC3568309

[60] Kazi MI, Conrado AR, Mey AR, Payne SM, Davies BW. 2016. ToxR antagonizes H-NS regulation of horizontally acquired genes to drive host colonization. PLoS Pathog 12:e1005570. doi:10.1371/journal.ppat.1005570. 27070545 PMC4829181

[61] Fan F, Liu Z, Jabeen N, Birdwell LD, Zhu J, Kan B. 2014. Enhanced interaction of Vibrio cholerae virulence regulators TcpP and ToxR under oxygen-limiting conditions. Infect Immun 82:1676–1682. doi:10.1128/IAI.01377-13. 24491579 PMC3993381

[62] Park N-Y, Kim IH, Wen Y, Lee K-W, Lee S, Kim J-A, Jung K-H, Lee K-H, Kim K-S. 2019. Multi-factor regulation of the master modulator LeuO for the cyclic-(Phe-Pro) signaling pathway in Vibrio vulnificus. Sci Rep 9:20135. doi:10.1038/s41598-019-56855-4. 31882984 PMC6934829

[63] Bina XR, Taylor DL, Vikram A, Ante VM, Bina JE. 2013. Vibrio cholerae ToxR downregulates virulence factor production in response to cyclo(Phe-Pro). mBio 4:e00366-13. doi:10.1128/mBio.00366-13. PMC3760244 23982069

[64] Ramadurai S, Holt A, Krasnikov V, van den Bogaart G, Killian JA, Poolman B. 2009. Lateral diffusion of membrane proteins. J Am Chem Soc 131:12650–12656. doi:10.1021/ja902853g. 19673517

[65] Lucena D, Mauri M, Schmidt F, Eckhardt B, Graumann PL. 2018. Microdomain formation is a general property of bacterial membrane proteins and induces heterogeneity of diffusion patterns. BMC Biol 16:97. doi:10.1186/s12915-018-0561-0. 30173665 PMC6120080

[66] Lorent JH, Diaz-Rohrer B, Lin X, Spring K, Gorfe AA, Levental KR, Levental I. 2018. Author correction: structural determinants and functional consequences of protein affinity for membrane rafts. Nat Commun 9:1805. doi:10.1038/s41467-018-04164-1. 29717140 PMC5931578

[67] Bina J, Zhu J, Dziejman M, Faruque S, Calderwood S, Mekalanos J. 2003. ToxR regulon of Vibrio cholerae and its expression in vibrios shed by cholera patients. Proc Natl Acad Sci USA 100:2801–2806. doi:10.1073/pnas.2628026100. 12601157 PMC151421

[68] Cold Spring Harbor Laboratory Press. 2016. LB solid or liquid medium. Cold Spring Harbor Protoc 2016:pdb.rec088203. doi:10.1101/pdb.rec088203.

[69] Skorupski K, Taylor RK. 1996. Positive selection vectors for allelic exchange. Gene 169:47–52. doi:10.1016/0378-1119(95)00793-8. 8635748

[70] Amin Marashi SM, Rajabnia R, Imani Fooladi AA, Hojati Z, Moghim S, Nasr Esfahani B. 2013. Determination of ctxAB expression in Vibrio cholerae classical and El Tor strains using real-time PCR. Int J Mol Cell Med 2:9–13. 24551784 PMC3920520

[71] Isaacoff BP, Li Y, Lee SA, Biteen JS. 2019. SMALL-LABS: measuring single-molecule intensity and position in obscuring backgrounds. Biophys J 116:975–982. doi:10.1016/j.bpj.2019.02.006. 30846363 PMC6428939

[72] Liao Y, Schroeder JW, Gao B, Simmons LA, Biteen JS. 2015. Single-molecule motions and interactions in live cells reveal target search dynamics in mismatch repair. Proc Natl Acad Sci USA 112:E6898–E6906. doi:10.1073/pnas.1507386112. 26575623 PMC4687589

[73] Munkres J. 1957. Algorithms for the assignment and transportation problems. J Soc Ind Appl Math 5:32–38. doi:10.1137/0105003.

[74] Schmittgen TD, Livak KJ. 2008. Analyzing real-time PCR data by the comparative C(T) method. Nat Protoc 3:1101–1108. doi:10.1038/nprot.2008.73. 18546601

