# Supplementary figures and images for: Independent Promoter Recognition by TcpP Precedes Cooperative Promoter Activation by TcpP and ToxR
Source: mBio. 2021 Sep 7;12(5):e02213-21. doi: 10.1128/mBio.02213-21 (PMC8546550; doi:10.1128/mBio.02213-21)

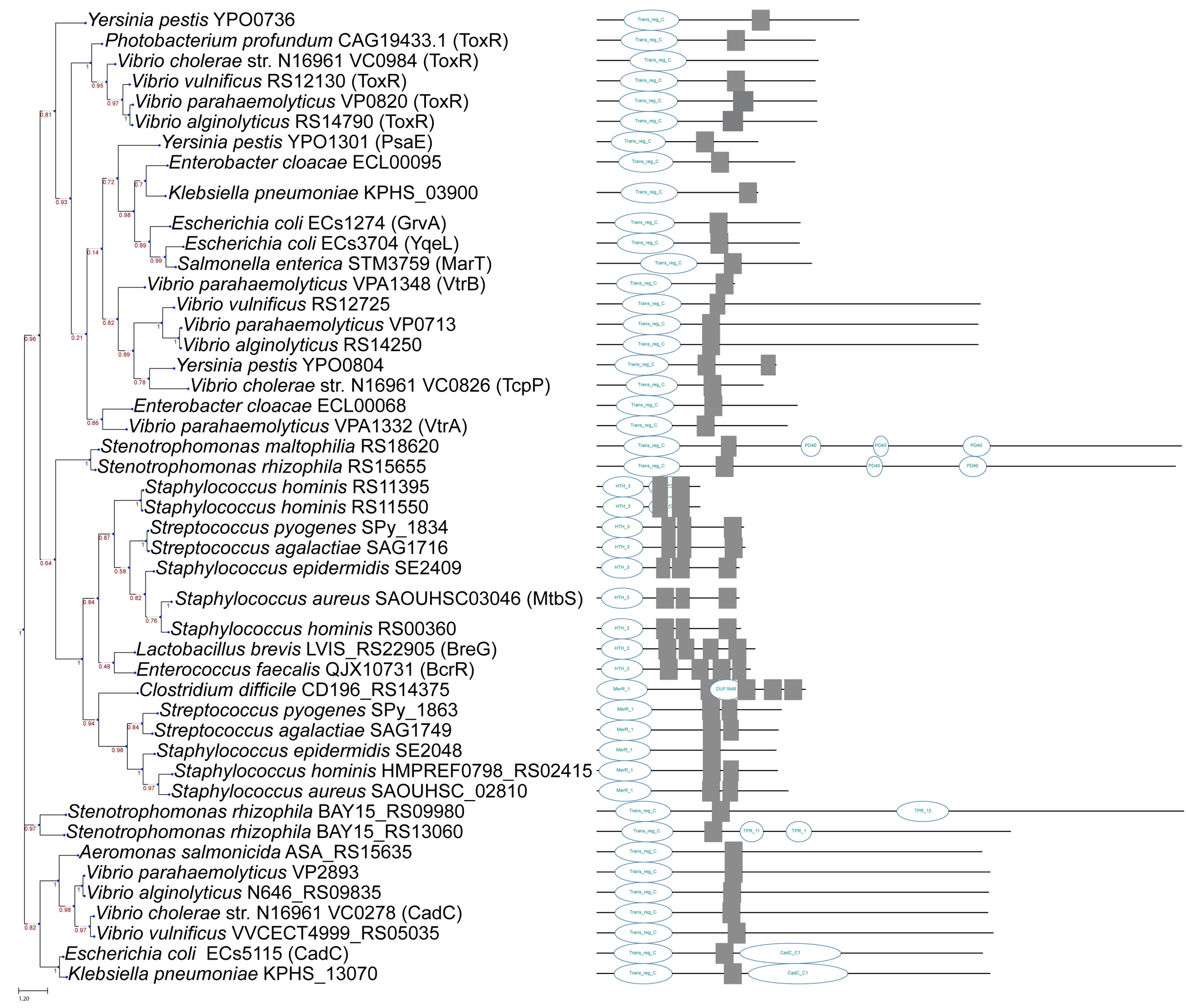

Supplement: FIG S1 [file mbio.02213-21-sf001.jpg]

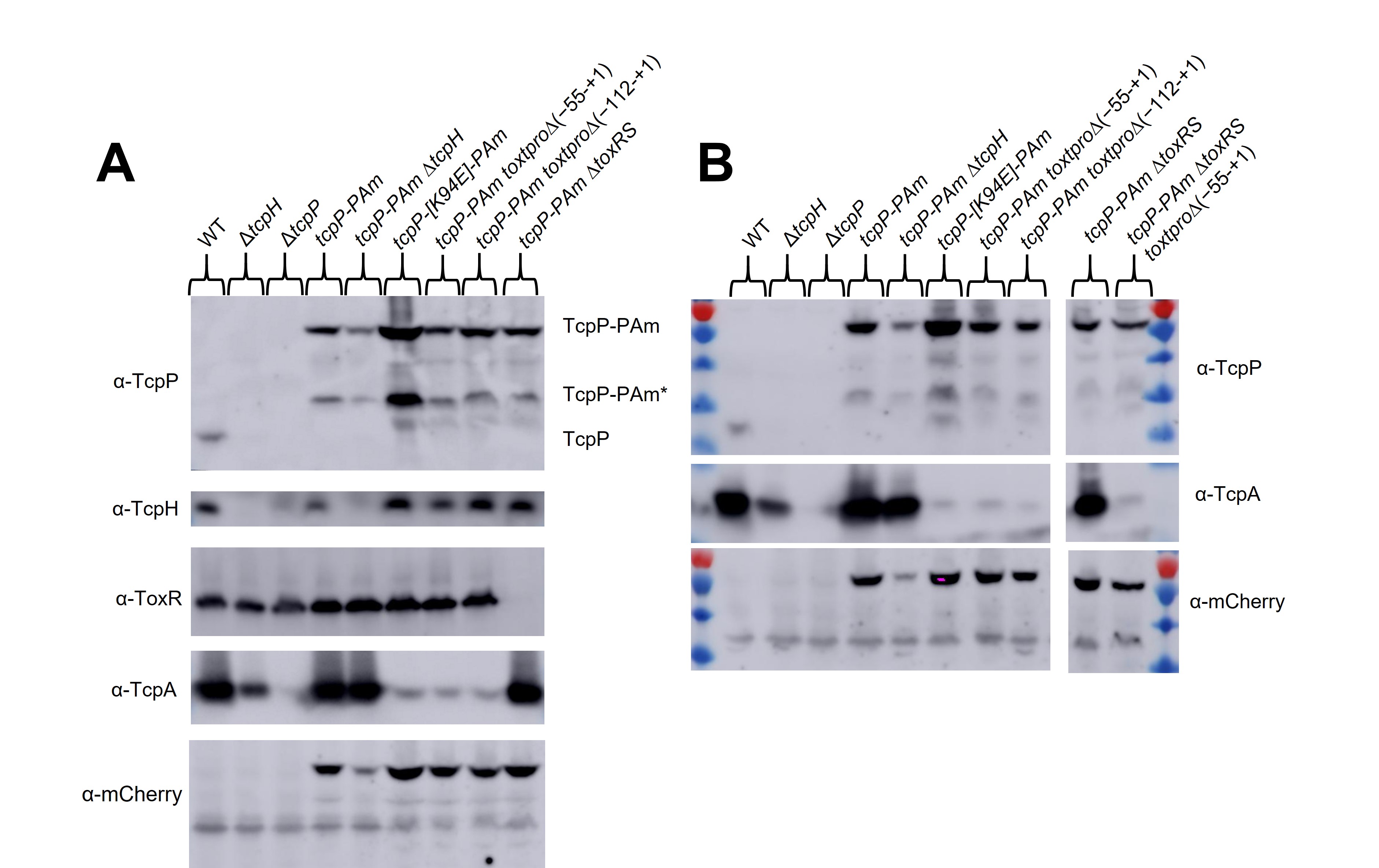

Supplement: FIG S2 [file mbio.02213-21-sf002.tif]

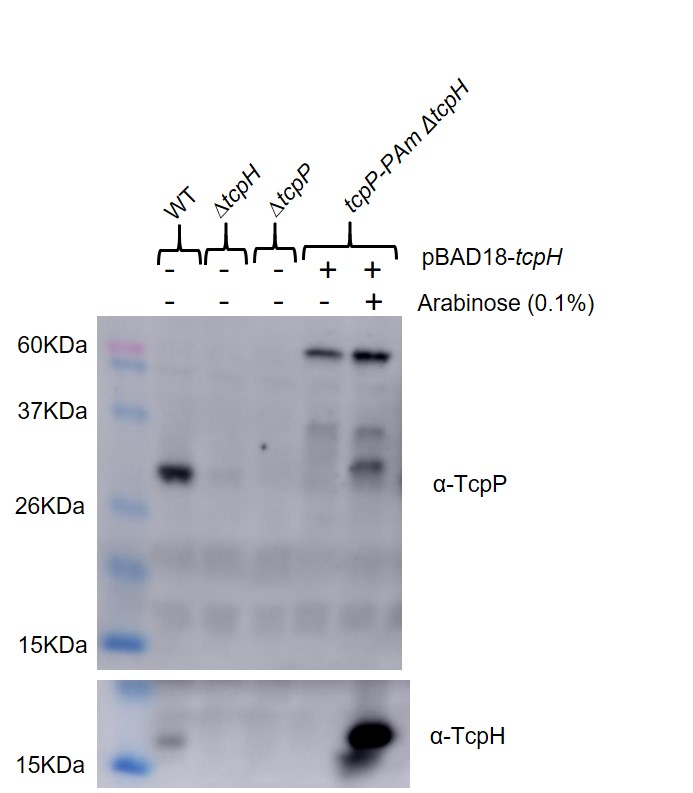

Supplement: FIG S3 [file mbio.02213-21-sf003.tif]

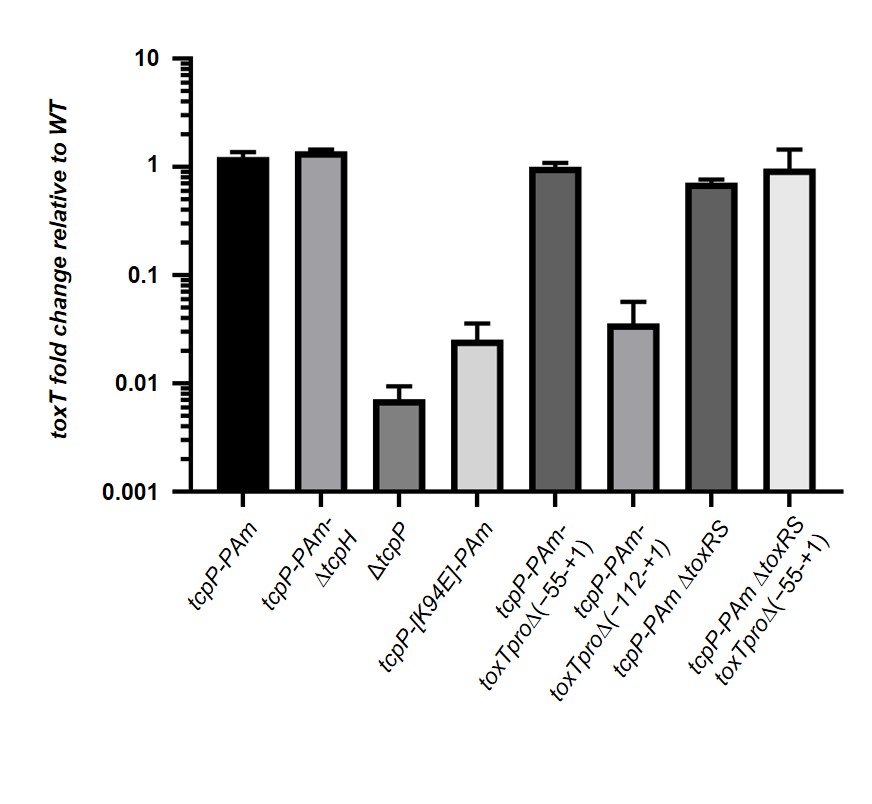

Supplement: FIG S4 [file mbio.02213-21-sf004.tif]

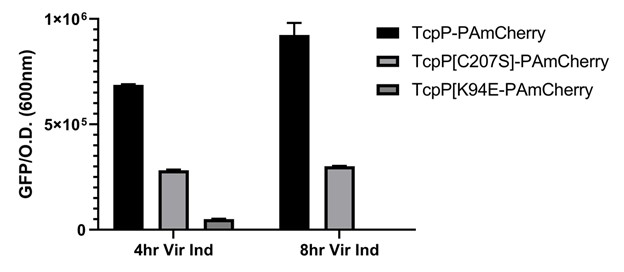

Supplement: FIG S5 [file mbio.02213-21-sf005.tif]

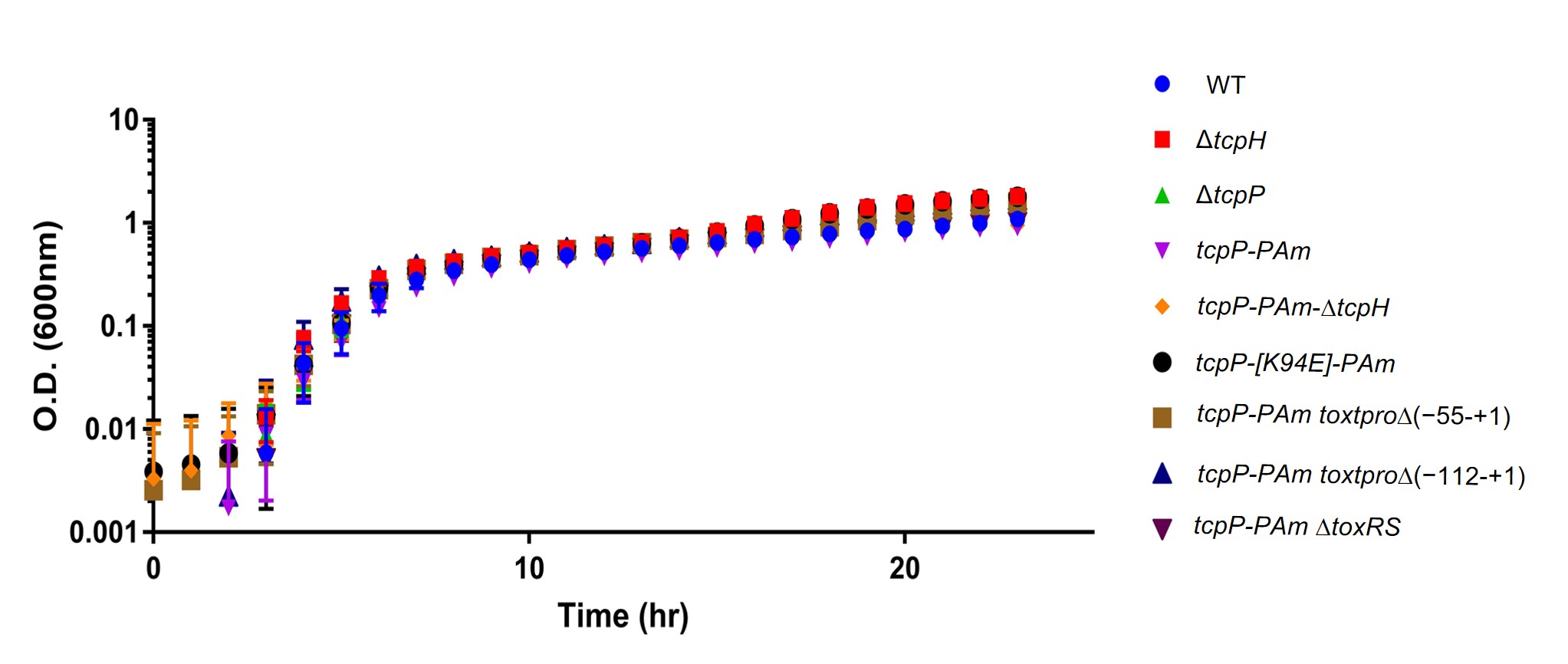

Supplement: FIG S6 [file mbio.02213-21-sf006.tif]

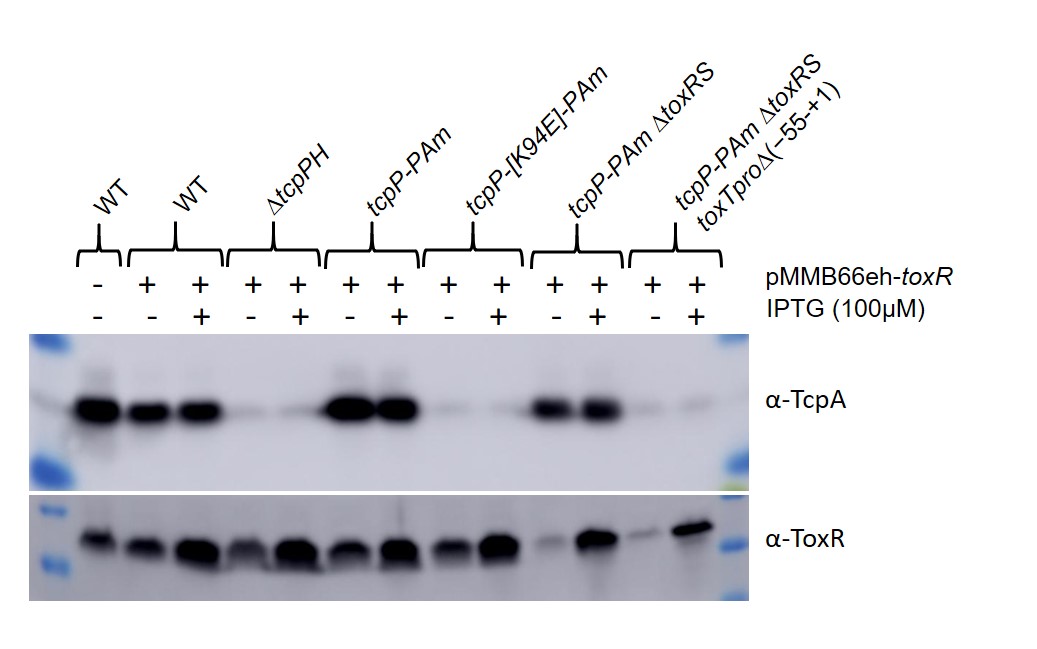

Supplement: FIG S7 [file mbio.02213-21-sf007.tif]

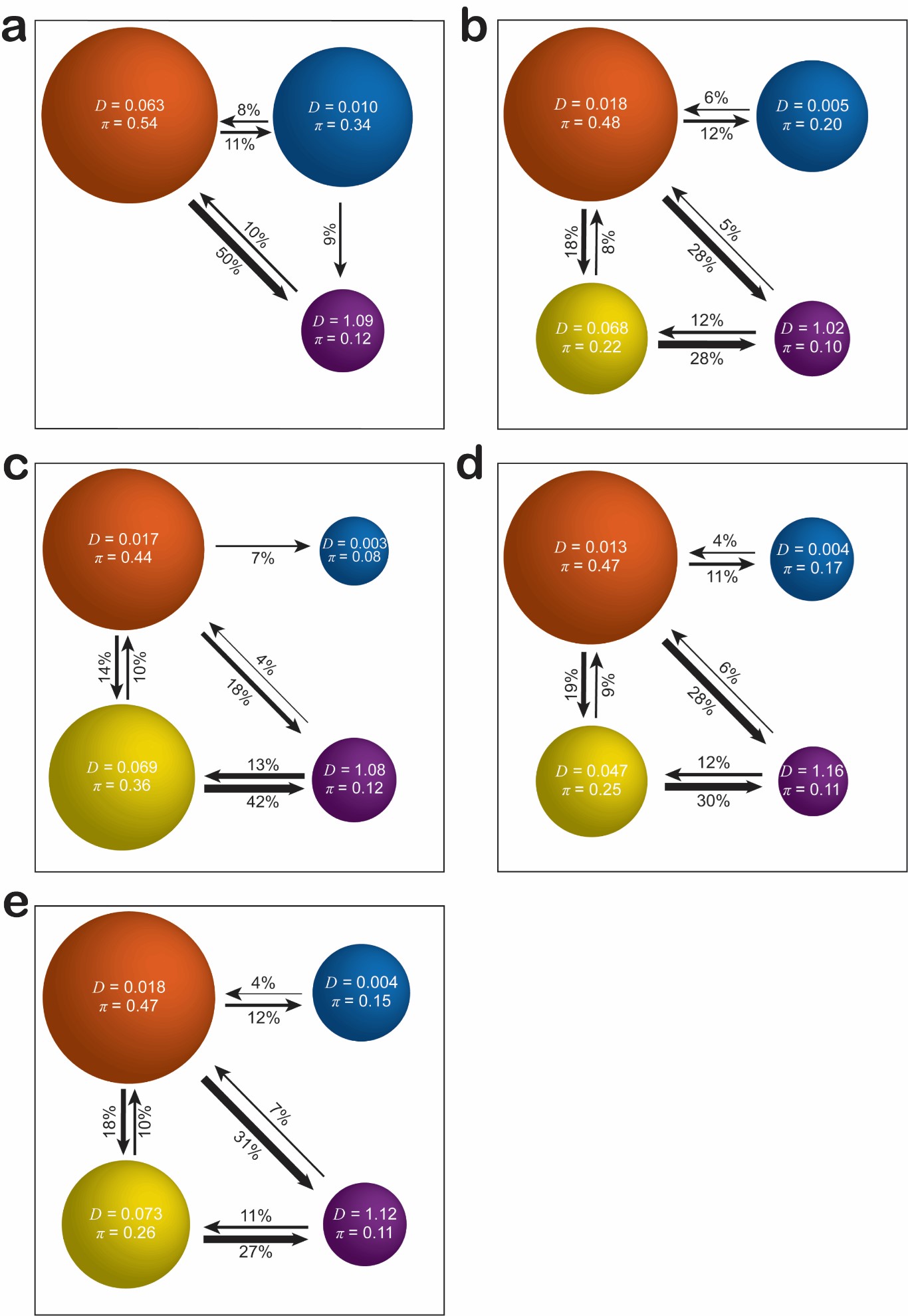

Supplement: FIG S8 [file mbio.02213-21-sf008.tif]

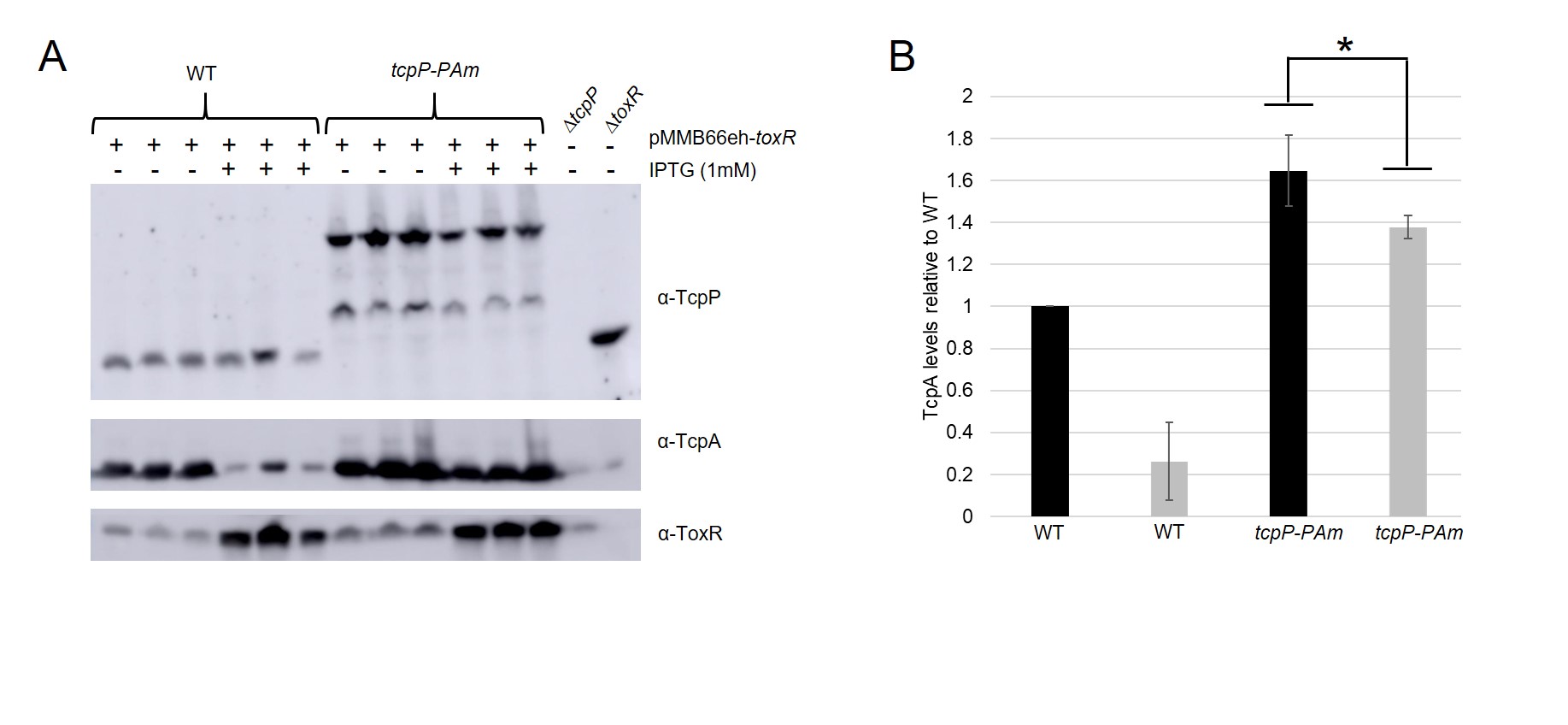

Supplement: FIG S9 [file mbio.02213-21-sf009.tif]
