# Supplementary material for: Independent Promoter Recognition by TcpP Precedes Cooperative Promoter Activation by TcpP and ToxR
Source: mBio. 2021 Sep 7;12(5):e02213-21. doi: 10.1128/mBio.02213-21 (PMC8546550; doi:10.1128/mBio.02213-21)
Supplement: TABLE S1 [file mbio.02213-21-st001.docx]

**Supplemental Table S1A:** Strain list.

| **Strain** | **Description** | **Reference** |
| --- | --- | --- |
| *V. cholerae* 0395 classical  biotype | Wild type | DiRita lab collection |
| *V. cholerae ∆tcpH* | Isogenic deletion | Beck, N.A., et. al. 2004. *Journal of bacteriology*,  *186*(24), p.8309. |
| *V. cholerae ∆tcpP* | Isogenic deletion | Häse, C.C. and Mekalanos, J.J., 1998. *Proceedings of the National Academy of Sciences*, *95*(2),  pp.730-734. |
| *V. cholerae ∆toxRS* | Isogenic deletion | DiRita lab collection |
| *V. cholerae tcpP-PAmCherry* | Isogenic construct; TcpP-PAmCherry (C-terminal fusion), native *tcpH* start codon and 3rd amino acid mutated (ATG to GTG and AAA to TAA respectively), and both ribosomal  binding site and coding sequence of | This study |

|  | *tcpH* cloned downstream of  PAmCherry. |  |
| --- | --- | --- |
| *V. cholerae tcpP-PAmCherry*  *∆tcpH* | Isogenic construct | This study |
| *V. cholerae tcpP-PAmCherry*  𝛥*toxRS* | Isogenic construct | This study |
| *V. cholerae tcpP-PAmCherry*  *∆toxRS* toxTpro*∆*(−55–+1) | Isogenic construct | This study |
| *V. cholerae tcpPK94E- PAmCherry* | Isogenic construct | This study |
| *V. cholerae tcpP-PAmCherry*  toxTpro*∆*(−55–+1) | Isogenic construct | This study |
| *V. cholerae tcpP-PAmCherry*  toxTpro*∆*(−112–+1) | Isogenic construct | This study |
| *V. cholerae tcpP-PAmCherry*  pMMB66eh-*toxR* | Isogenic construct | This Study |
| *E. coli* ET12567 *∆dapA* | Cloning vector recipient | Allard, N., et. al. 2015. Canadian journal of microbiology, 61(8),  pp.565-574. |

| *E. coli* ET12567 *∆dapA*  pKAS32-(empty vector) | Plasmid vector strain | Skorupski, K. and Taylor, R.K., 1996.  *Gene*, *169*(1),  pp.47-52. |
| --- | --- | --- |

**Supplementary Table S1B:** Primer list. Kpn1-HiFi restriction sites were included in forward primers and Xba1 restriction sites were included in all reverse primers to provide homology between insert and vector sequences.

| **Description** | **Sequence** |
| --- | --- |
| pKAS-TcpP promoter FW | ctaacgttaacaaccggtacTTTCGAGTGATAGAAAAAG  G |
| pKAS-TcpP FW | ctaacgttaacaaccggtacATGGGGTATGTCCGCGTG |
| TcpP-PAmCherry FW | atgcactaaaaatATGGTGAGCAAGGGCGAGGA |
| TcpP-PAmCherry RV | ccttgctcaccatATTTTTAGTGCATTCTAATGTCTTCT GTTC |
| TcpH-PAmCherry FW | ctaatgtcttCTTGTACAGCTCGTCCATGC |
| TcpH-PAmCherry RV | gctgtacaagAAGACATTAGAATGCACAAAAAATTAA AAG |
| Downstream TcpH-PAmCherry RV | tcatgataagaccCTTGTACAGCTCGTCCATGCC |
| Downstream TcpH-PAmCherry FW | cgagctgtacaagGGTCTTATCATGAGCCGCCTAG |
| pKAS-downstream TcpH RV | aaatttgcgcatgctagctatagttCTTGGTCTTTTTTAGATA ACGTAAGC |
| TcpPK94E RV | GATCAACGTCTCATGTTCATC |
| TcpPK94E FW | GATGAACATGAGACGTTGATC |

| *toxTpro ∆*(−55–+1) RV | tcccaatcatATCTTAAAATCGAAGTTAATATAAAACT  AC |
| --- | --- |
| *toxTpro ∆*(−55–+1) FW | gattttaagatATGATTGGGAAAAAATCTTTTC |
| pKAS-*toxTpro ∆*(−112–+1) FW | ctaacgttaacaaccggtacGTTGGTGGTGTTCCAGATA ATAC |
| *toxTpro ∆*(−112–+1) RV | ttcccaatcaGTATTACATAAGAAAAACATAAAGTAA CTCATG |
| *toxTpro ∆*(−112–+1) FW | tatgtaatacTGATTGGGAAAAAATCTTTTC |
| pKAS-*toxTpro ∆*(−112–+1) RV | tgcgcatgctagctatagttATCATCAGTAATAAATATAGA GTTATATTTTTTTTC |
| *recA* FW | ATTGAAGGCGAAATGGGCGATAG |
| *recA* RV | TACACATACAGTTGGATTGCTTG AGG |
| *toxT* FW | ACTGATGATCTTGATGCTATGGAG |
| *toxT* RV | CATCCGATTCGTTCTTAATTCACC |
